# MALDI TIMS IMS Reveals Ganglioside Molecular Diversity within Murine *S. aureus* Kidney Tissue Abscesses

**DOI:** 10.1021/jasms.4c00089

**Published:** 2024-07-25

**Authors:** Katerina V. Djambazova, Katherine N. Gibson-Corley, Jeffrey A. Freiberg, Richard M. Caprioli, Eric P. Skaar, Jeffrey M. Spraggins

**Affiliations:** † Department of Cell and Developmental Biology, Vanderbilt University, Nashville, Tennessee 37232, United States; ‡ Mass Spectrometry Research Center, Vanderbilt University, Nashville, Tennessee 37232, United States; § Department of Pathology, Microbiology, and Immunology, Vanderbilt University Medical Center, Nashville, Tennessee 37232, United States; ∥ Vanderbilt Institute for Infection, Immunology and Inflammation, Vanderbilt University Medical Center, Nashville, Tennessee 37232, United States; ⊥ Division of Infectious Diseases, Department of Medicine, Vanderbilt University Medical Center, Nashville, Tennessee 37232, United States; # Department of Biochemistry, Vanderbilt University, Nashville, Tennessee 37232, United States; ∇ Department of Pharmacology, Vanderbilt University, Nashville, Tennessee 37232, United States; ○ Department of Medicine, Vanderbilt University, Nashville, Tennessee 37232, United States; ◆ Department of Chemistry, Vanderbilt University, Nashville, Tennessee 37232, United States; ¶ Vanderbilt Institute for Chemical Biology, Vanderbilt University, Nashville, Tennessee 37232, United States

**Keywords:** Gangliosides, Isomers, Staphylococcus aureus, Imaging mass spectrometry, Matrix-assisted laser desorption/ionization, Trapped ion mobility spectrometry, Ion mobility mass spectrometry

## Abstract

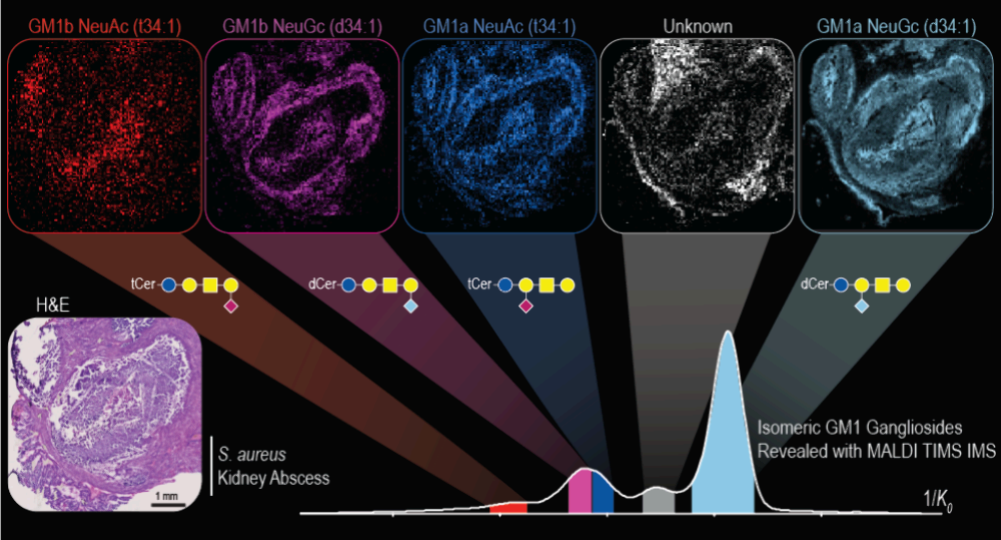

Gangliosides play important roles in innate and adaptive immunity. The high degree of structural heterogeneity results in significant variability in ganglioside expression patterns and greatly complicates linking structure and function. Structural characterization at the site of infection is essential in elucidating host ganglioside function in response to invading pathogens, such as *Staphylococcus aureus* (*S. aureus*). Matrix-assisted laser desorption/ionization imaging mass spectrometry (MALDI IMS) enables high-specificity spatial investigation of intact gangliosides. Here, ganglioside structural and spatial heterogeneity within an *S. aureus*-infected mouse kidney abscess was characterized. Differences in spatial distributions were observed for gangliosides of different classes and those that differ in ceramide chain composition and oligosaccharide-bound sialic acid. Furthermore, integrating trapped ion mobility spectrometry (TIMS) allowed for the gas-phase separation and visualization of monosialylated ganglioside isomers that differ in sialic acid type and position. The isomers differ in spatial distributions within the host–pathogen interface, where molecular patterns revealed new molecular zones in the abscess previously unidentified by traditional histology.

## Introduction

*Staphylococcus aureus* (*S. aureus*) is a widespread commensal Gram-positive bacterium and pathogen.^1−3^ It is the leading cause of skin and soft tissue infections and can result in more serious complications, including bacteremia, pneumonia, and osteomyelitis.^4^ Staphylococcal invasion typically leads to soft-tissue abscess formation, where the bacteria organize into staphylococcal abscess communities (SACs), surrounded by severe inflammation of neighboring tissues.^3−5^ The mature abscess is a highly heterogeneous environment with a diverse subset of host- and pathogen-derived molecules.^5,6^ Traditional histological approaches such as stained microscopy and immunohistochemistry (IHC) can reveal the abscess architecture and the spatial distributions of specific targets within the tissues.^3^ However, detailed molecular information cannot be gleaned from these techniques, and more advanced analytical approaches, such as imaging mass spectrometry (IMS) are necessary to provide molecular and spatial context.^7^

Matrix-assisted laser desorption/ionization (MALDI) IMS allows for direct visualization of metabolites, lipids, peptides, and other biomolecules from tissue sections.^7−9^ In a typical MALDI IMS experiment, tissue sections are thaw-mounted onto conductive glass slides and coated with a UV-absorbing matrix. A laser is rastered across the sample to produce individual mass spectra at each ablation position. Molecular images are generated by plotting the ion intensities as a heat map across all ablation spots (pixels).^10^ MALDI IMS is a minimally destructive approach, which allows for downstream histology to be performed.^11,12^ In this manner, histology and molecular information are correlated, allowing for a thorough investigation of the spatio-molecular characteristics of *S. aureus* infections.^13−17^ With these multimodal approaches we can visualize and study molecules involved in host–pathogen interactions.^18−21^

Gangliosides (acidic glycosphingolipids) are immune system modulators that can alter both innate and adaptive immune functions.^20−22^ They reside primarily in the cellular plasma membrane, where the ceramide anchors the molecule to the membrane and the glycan protrudes outward from the cell.^23^ At the cell surface, gangliosides can interact with other molecules and regulate the activity of proteins.^19,20,24^ Gangliosides can also be unintentional targets for microbial adhesion, where viruses, bacteria, and bacterial toxins bind to the carbohydrate of gangliosides on host cell surfaces.^18−20,22,25^ Elucidating the distinct functions of gangliosides in response to invading pathogens is particularly challenging due to their vast structural diversity. Thus, comprehensive structural information on gangliosides at the site of infection is a critical first step in exploring ganglioside function in immune response and the interaction between host and pathogen.

**Figure 1:**
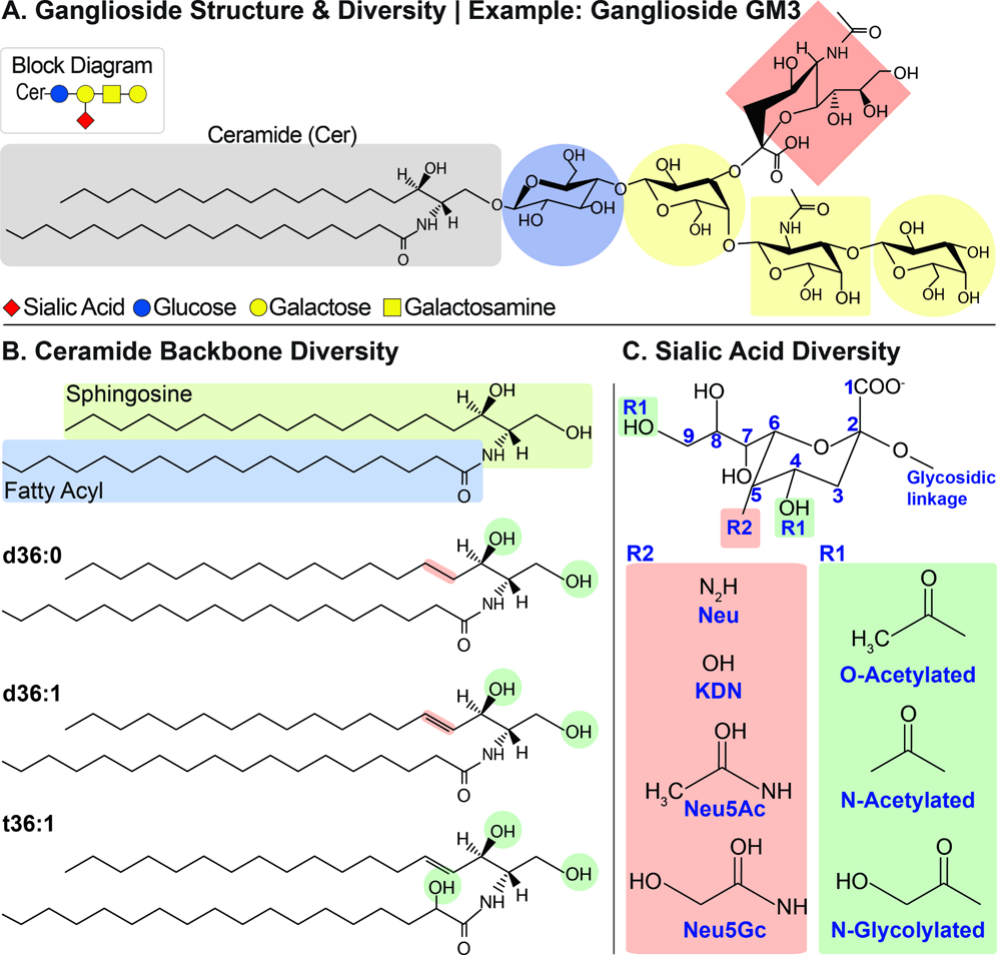
Ganglioside molecular structure (GM3) example (A) highlights common diversities in the ceramide backbone (B) and common sialic acids and their alterations (C); Neu, neuraminic acid; KDN, deaminated neuraminic acid; NeuAc, *N*-acetylneuraminic acid; NeuGc, *N*-glycolylneuraminic acid.

Structurally, the ganglioside molecule can be divided into two functional units–a hydrophilic oligosaccharide headgroup carrying sialic acid(s) and a hydrophobic ceramide moiety. (Figure 1A). The ceramide moiety of the gangliosides is composed of a sphingosine and a fatty acyl chain (Figure 1B). The fatty acyls in gangliosides generally range from 16 to 30 carbons, with varying degrees of unsaturation. The most common sphingoid bases are d18:1 and d20:1, where 18 and 20 refer to the number of carbons in the chain, 1 indicates the presence of a single double bond, and *d* (“di” - two) denotes the number of hydroxyl groups on the sphingoid base. Other sphingoid bases present in eukaryotes include, but are not limited to, d18:0, d20:0, d16:0, and t18:1 (“*tri*” - three hydroxyl groups). Both hydrocarbon length and the number of double bonds of the ceramide can dictate biological function. The hydrophilic oligosaccharide headgroup is comprised of multiple saccharide units, including glucose (Glc), galactose (Gal), galactosamine (GalNAc), and sialic acids (Figure 1).^26^ Gangliosides are synthesized by the stepwise addition of monosaccharides to the glucose-ceramide (GlcCer) unit (Figure S1). After the addition of Gal, the lactosylceramide (LacCer) serves as a building block for all subsequent gangliosides and is a branching point in the synthesis. From there, the oligosaccharide chain can be elongated by the addition of monosaccharides, creating the o-series gangliosides (GA2, GA1, GM1b). Alternatively, the sequential addition of sialic acids to LacCer by sialyltransferases form gangliosides GM3, GD3, GT3, etc. Each of these serves as a precursor for more complex gangliosides, belonging to the a-, b-, and c-series, respectively. The letter refers to the number of sialic acids bound to the internal galactose unit, resulting in o-(zero), a-(one), b-(two), c-(three) series gangliosides.^23,26−29^ The differences in the oligosaccharide chain and the quantity/position of sialic acids greatly increase the number of possible structures and their biological roles.

Finally, negatively charged sialic acids are essential components of the ganglioside molecule. They alone can influence ganglioside function, particularly in pathogen recognition and cell infiltration.^30,31^ Although all sialic acids share the same 9-carbon carboxylated sugar, there are numerous structural differences and modifications that can be present. The most common ganglioside-bound sialic acids are *N*-acetylneuraminic acid (NeuAc) and *N*-glycolyneuraminic acid (NeuGc),^32^ where common modifications include O-acetylation, N-acetylation, and N-glycolylation (Figure 1C). The diversity of sialic acid structures at various sialylation sites greatly increases the number of possible structures. For example, although ∼200 ganglioside structures have been described in the literature, considering the known diversity of the ceramide and the glycan headgroup, it is hypothesized that over 3000 unique gangliosides exist.^28,29^

Mass spectrometry has been at the forefront of ganglioside analysis, where liquid chromatography (LC)-based studies have provided detailed structural information.^33−35^ MALDI IMS has been used to investigate the spatial distributions of these analytes^36−39^ and gas-phase separations, such as with ion mobility, have provided further structural characterization in an imaging context, however, with limited specificity.^40,41^ Recently, we demonstrated MALDI trapped ion mobility spectrometry (TIMS) IMS for the separation and visualization of a- and b-series GD1 isomers within rat brain and spinal cord tissues.^40−42^ Briefly, TIMS separations are carried out in the first vacuum stage of a mass spectrometer in an augmented ion funnel composed of an entrance funnel, TIMS tunnel, and exit funnel.^43−45^ Propelled by a carrier gas, ions are accumulated, trapped, and separated by an electric field.^46−48^ To elute trapped ions, the electric field gradient is gradually reduced, releasing ions with ascending mobilities. TIMS IMS is capable of high resolving power separations (>200 in 50–500 ms), ideal for addressing structural heterogeneity and isomerism prevalent in this molecular class.^42,45,46,48,49^

Here, MALDI TIMS IMS of *S. aureus*-infected mouse kidney was used for in-depth structural characterization of gangliosides within an inflammatory lesion. The structural heterogeneity of gangliosides including ceramide composition, oligosaccharide chain, as well as sialic-acid positional information, was characterized. Furthermore, ganglioside isomers were resolved by TIMS separations, and their distinct distributions within the abscess were visualized.

## Methods

### Materials

2′,5′-Dihydroxyacetophenone (DHA), hematoxylin, eosin, and ammonium sulfate were purchased from Sigma-Aldrich (St. Louis, MO, USA). HPLC-grade acetonitrile, and ethanol, were purchased from Fisher Scientific (Pittsburgh, PA, USA). Indium tin oxide-coated slides (CG-81IN-S115) were purchased from Delta Technologies, Limited (Loveland, CO, USA).

### Murine Model of *S. aureus* Infection

Female C57BL/6J mice (6–8 weeks old) (Jackson Laboratory) were retro-orbitally infected with 1 × 10^7^ colony forming units (CFU) of S*taphylococcus aureus* Newman in 100 μL of sterile phosphate-buffered saline as previously described.^13^ Following infection, the mice were euthanized 10 days postinfection (DPI). The organs were removed, immediately frozen on a bed of dry ice, and stored at −80 °C until further processing. All animal experimental protocols were reviewed and approved by the Vanderbilt University Institutional Animal Care and Use Committee and were in compliance with institutional policies, NIH guidelines, the Animal Welfare Act, and American Veterinary Medical Association guidelines on euthanasia.

### Sample Preparation

Infected (10 DPI) and control mouse kidney sections were cryosectioned to 10 μm thickness using a CM3050 S cryostat (Leica Biosystems, Wetzlar, Germany) and thaw-mounted onto conductive indium tin oxide-coated glass slides (Delta Technologies, Loveland, CO, USA). Matrix (2′,5′- DHA with 62.5 μM ammonium sulfate in 60% ethanol–water) was applied using a robotic sprayer (M5 Sprayer, HTX Technologies, NC, USA) for a final matrix density of 1.48 μg/mm^2^ (Table S1). Immediately following MALDI IMS, the matrix was removed from the sample using 100% ethanol and rehydrated with graded ethanol and H_2_O. Tissues were stained using an H&E stain. Brightfield microscopy was obtained at 20× magnification using a Zeis AxioScan Z1 slide scanner (Carl Zeiss Microscopy GmbH, Oberkochen, Germany).

### MALDI TIMS IMS

All experiments were carried out on a prototype MALDI timsTOF fleX mass spectrometer (Bruker Daltonics, Bremen, Germany).^45^ Data were acquired in negative ionization mode (*m*/*z* 1,000–3,000) at 50 μm (45 μm beam scan) spatial resolution with ∼50% laser power at 10 kHz, 400 shots per pixel, and 35,680 (infected) and 19,694 (control) pixels per sample. MALDI TIMS data were collected over a 1/*K*_*0*_ range of 1.50–2.45 V·s/cm^2^ (T3 Ramp: 230.5–124.7 V) with a ramp time of 450 ms, resulting in a scan rate (Sr) of 0.24 V/ms. The following parameters were kept constant across all imaging experiments: ESI dry gas temperature, 100 °C; Ion transfer time, 120 μs; prepulse storage time, 12 μs; collision RF, 3500 Vpp; TIMS funnel 1 RF, 450 Vpp; TIMS funnel 2 RF , 400 Vpp; multipole RF, 400 Vpp, collision cell entrance voltage, −200 V; MALDI deflection plate, 90 V. The source pressure was set to ∼2.35 mbar, to access higher 1/*K*_*0*_ ranges. Both MS and TIMS calibrations were performed using an Agilent ESI-L tuning mixture.

### Identification and Data Analysis

Serial tissue sections were analyzed for further structural investigation. Fourier Transform-Ion Cyclotron Resonance (FT-ICR) MS (15T SolariX, Bruker Daltonics, Bremen, Germany) with ultrahigh-spectra resolution (∼200,000 at *m*/*z* 1544.86) provided exact mass measurements and eliminated the possibility of isobaric interferences. On-tissue MALDI TIMS MS/MS was collected from a small region of a serial tissue section for at least one representative species of each ganglioside class. Experimental details, such as sampled area (number of pixels), CID voltage, and isolation window width, are provided in the relevant supplemental figure captions. Table S2 summarizes all characteristic fragment ions used to make identification in this study. We retained ganglioside identifications if the mass error was <5 ppm by MALDI TIMS MS or FTICR MS; if the mass error was >5 ppm and no on-tissue fragmentation was collected for the species, data were removed from the final list of identified gangliosides. MALDI TIMS IMS data were analyzed and visualized using DataAnalysis and SCiLS (Bruker Daltonics, Bremen, Germany), respectively. All ion images were generated using a peak’s centroid *m*/*z* ± 10 mDa; images generated with both *m*/*z* and ion mobility information were generated with the following boundaries *m*/*z* ± 10 mDa and peak 1/*K*_*0*_ ± 0.007 V•s/cm^2^.

## Results and Discussion

### Molecular and Structural Diversity within Murine Kidney Tissue Abscess

**Figure 2:**
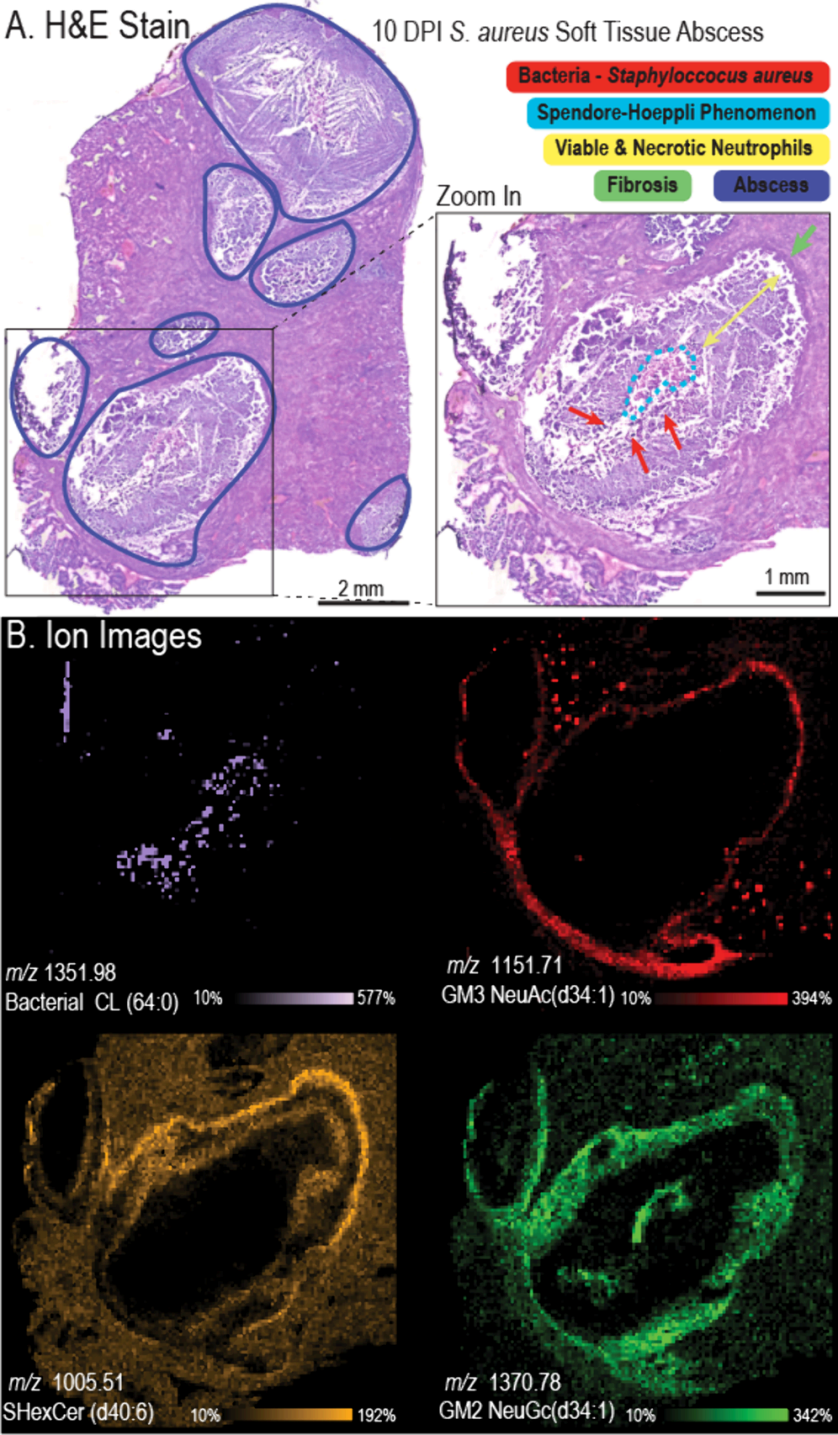
H&E Stain of 10 DPI *S. aureus*-infected mouse kidney section reveals several abscess lesions (blue boundaries) within the kidney section. A zoom-in of the lesion reveals abscess structures including a dark stained fibrous capsule (green), bacterial staphylococcal abscess communities (red arrows), a zone of healthy and dead immune cells (yellow arrow), and an intensely eosinophilic region, tentatively identified as the Splendore-Hoeppli phenomenon (bright blue dotted line) (A). Negative ion mode MALDI IMS images highlight different structures within the lesion (B).

To investigate molecular heterogeneity at the host–pathogen interface, a 10 DPI mouse kidney tissue section was analyzed using MALDI TIMS IMS. Data were collected in negative ionization mode, where all analytes were detected as deprotonated [M-H]^−^ ions. Average mass spectra comparing control and 10 DPI mouse kidney tissue sections can be seen in Figure S2, and the ion mobility heat map of the average mass spectrum of the 10 DPI mouse kidney data can be seen in Figure S3. An H&E stain of the infected sample was performed following IMS, and the microscopy image was histopathologically assessed to annotate known abscess morphology features by a board-certified Veterinary Pathologist (Figure 2A).

The SACs were identified at the center of the abscess (red arrows). The primary immune cells observed in the abscess were neutrophils, with degenerate neutrophils (pus) found near the bacterial colonies, and a mixture of necrotic and viable immune cells (most commonly neutrophils) found further from the abscess center (yellow arrow). The intensely eosinophilic material (bright blue boundary) is Splendore-Hoeppli phenomenon (reaction).^53−,100^ This phenomenon is an *in vivo* formation of eosinophilic material, which has been observed around microorganisms like bacteria and fungi. While its’ nature and mechanism are not well understood, it is thought to be a deposition of antigen–antibody complexes and debris from the host inflammatory cells.^53,100^ Finally, the microscopy also revealed a dark-stained fibrous capsule layer (fibrosis) at the outer abscess border (green), separating the abscess from the normal host tissue.

MALDI IMS data revealed a diverse molecular landscape within the abscess, as shown in Figure 2B. The infected mouse was sacrificed during late-stage infection (10 DPI), therefore the bacteria occupy a relatively small area of the lesion, as the majority of the abscess is made up of infiltrating immune cells and cellular debris. Bacterial cardiolipin CL (64:0) (*m*/*z* 1351.98, pink) revealed the spatial distributions of the bacteria within the abscess, and GM3 NeuAc (d34:1) (*m*/*z* 1151.71, red) colocalized with the fibrous capsule and the glomeruli.^15,50,51^ While the distributions of these ions correlate well with known anatomical features, other ions reveal molecular heterogeneity not evident in the microscopy. For example, sulfated hexosylceramide SHexCer (d40:6) (*m*/*z* 1005.51, yellow) and GM2 NeuGc (d34:1) (*m*/*z* 1370.78, green) reveal distinct layers within the zone of healthy/necrotic immune cells, where the histology revealed a largely homogeneous layer. GM2 NeuGc (d34:1) also colocalized to the intensely eosinophilic region peripheral to the bacteria, identified as the Splendore-Hoeppli phenomenon. This reaction has been previously observed by histological assessment and is thought to be made up of glycoproteins, lipids, and calcium derived from host leukocytes.^53,52^ However, the molecular makeup of Splendore-Hoeppli remains unknown; approaches like MALDI IMS offer a unique insight into the molecular landscape of this unique morphology.

### Ganglioside Heterogeneity within Murine *S. aureus* Soft Tissue Abscesses

**Figure 3:**
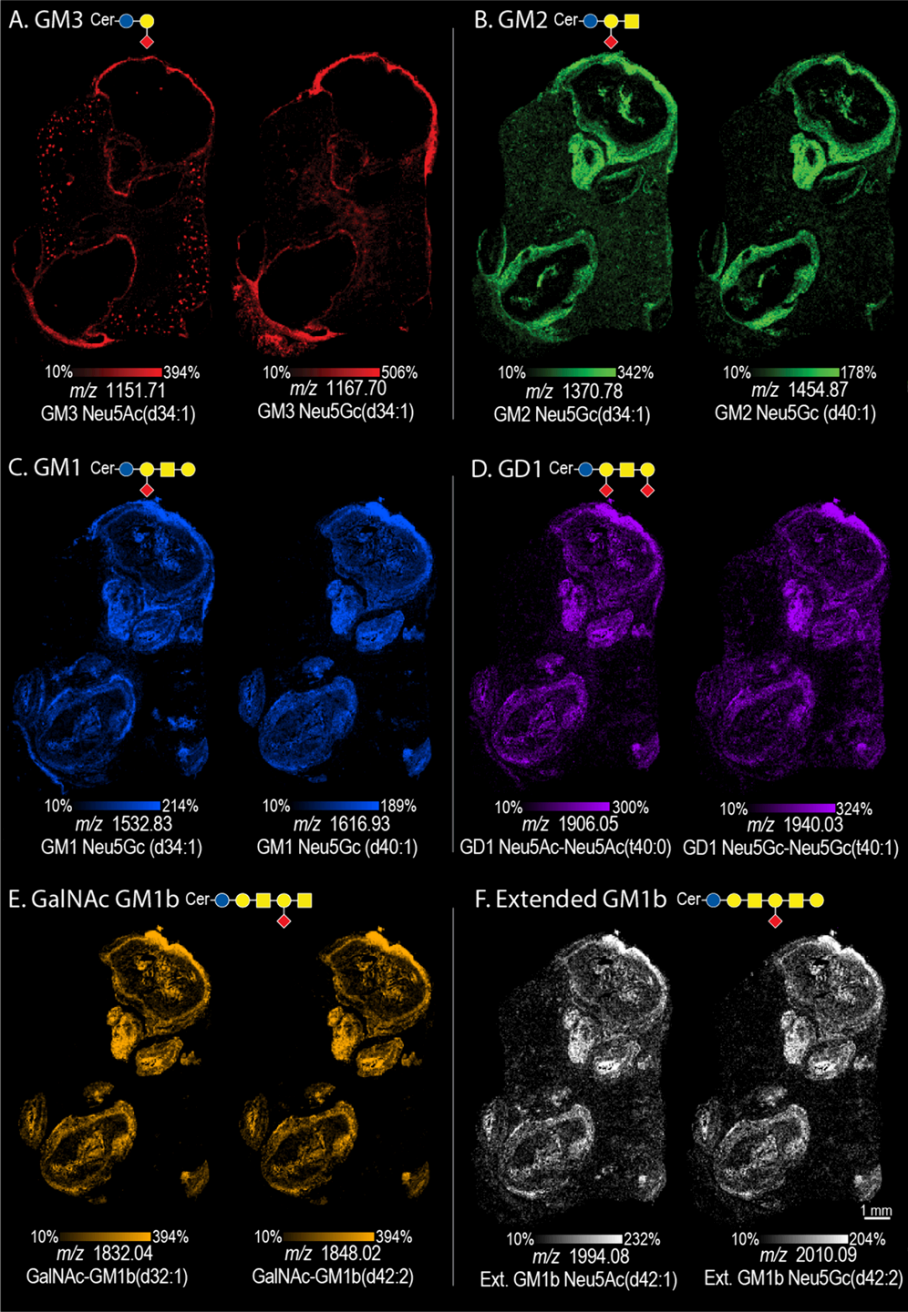
MALDI TIMS IMS shows structural and spatial diversity within a 10 DPI *S.aureus*-infected mouse kidney section across ganglioside classes: GM3 (A), GM2 (B), GM1 (C), GD1 (D), GalNAc-GM1b (E), and extended GM1b (F). No mobility information was used to generate the ion images.

MALDI TIMS IMS was performed in negative ionization mode; all gangliosides were detected as deprotonated [M-H]^−^ ions. Six different classes of gangliosides, including monosialylated (GM1, GM2, and GM3) and disialylated (GD1) were detected within the *S. aureus*-infected mouse kidney section (Figures 3, S2, and S3). Rare GalNAc-GM1b and extended series-GM1b gangliosides were also observed. These species have only been observed in a few immune cell types, including T-cells, B-cells, and Macrophages; however, their functions and abundance in other immune cells are not well understood.^21,55^

GM3 species are the simplest a-series gangliosides, composed of two carbohydrate units and a single sialic acid. A total of 11 GM3 species were detected in the infected kidney, where the ceramide chains ranged from C34 to C42 in length (Table S3, Figure 3A). Monounsaturated dihydroxylated ceramide chains were most common, and no unsaturated species were detected. In terms of sialic acid diversity, both NeuAc and NeuGc sialic acids were detected. GM3s were the only ganglioside species observed in both the control and infected tissue; a total of 8 GM3 species were observed in the control (Table S3, Figure S4). For example, *m*/*z* 1151.71, identified as GM3 NeuAc(d34:1), localized to the fibrous capsule of the abscess and the glomeruli (kidney filtering units). All GM3s localized to the fibrous capsule and were not detected in the intra-abscess region.

GM2s are synthesized from GM3s with the addition of GalNAc to the oligosaccharide chain to form a trisaccharide headgroup with a single sialic acid residue. A total of four GM2 species were detected in the infected tissue sample (Figure 3B, Table S4, Figure S5) All GM2s had a dihydroxylated ceramide backbone, and only NeuGc sialic acids were detected. An example on-tissue MALDI MS/MS spectrum is shown for GM2 NeuGC (d34:1) (*m*/*z* 1370.78) in Figure S6. All ions localized to the outer abscess layers, and signals were also observed neighboring the center of the abscess (Splendore-Hoeppli phenomenon). While the exact role of GM2s within infection processes is not completely understood,^21^ it has been noted that GM2s can serve as receptors for bacterial toxin binding.^18^

The most abundant ganglioside class detected in the infected mouse kidney sample was GM1. GM1s contain a tetra-saccharide core and a single sialic acid. GM1s have high structural diversity resulting in several possible isomeric structures. In this example, 17 different GM1s were differentiated by mass alone, and an additional 8 were revealed by TIMS analysis (Figure 3C, Tables S5 and S7). The ceramide chain compositions ranged from C34 to C42, where fully unsaturated, di- and tri-unsaturated ceramide chains were observed. Dihydroxylated ceramides were most common, however, several gangliosides with trihydroxylated chains were also detected. GM1s were detected throughout various intra-abscess structures and in the surrounding immune cell infiltrates.

GD1s are disialylated gangliosides, comprised of a tetrasaccharide carbohydrate chain with two sialic acids. Numerous GD1 species, as highlighted in Table S6 and Figure S8, were detected in the *S. aureus*-infected mouse kidney (Figure 3D). Polysialylated species, such as GD1, can be particularly challenging to analyze, due to the in-source fragmentation of the labile sialic acid bond, as well as the high number of possible isomeric structures. Therefore, confident identification of all GD1s could not be achieved. More complex extended o-series gangliosides, such as GalNAc-GM1b, and extended series GM1b species are synthesized from GM1b gangliosides by the sequential addition of GalNAc, and Gal-GalNAc, respectively. Here, 16 different GalNAc-GM1b and three extended series GM1b gangliosides were detected (Table S7, Figure S9). Structures were confirmed with on-tissue MS/MS, where an example fragmentation spectrum for GalNAc-GM1b (d42:1) is highlighted in Figure S10. The spatial distributions of these gangliosides can be seen in Figure 3E and F. While Sarbu et al.^101^ recently described GalNAc-GD1 isomers in cerebrospinal fluid, to our knowledge, GalNAc- and extended GM1b gangliosides have not been detected or visualized with MALDI IMS to date. Little is known about the biological function of extended series gangliosides. They have been reported as part of T-cell membranes, where they have been linked to T-cell differentiation and activation.^20,21,24^ However, our results indicate that they may also be crucial components of other immune cells since infection would primarily activate innate immune cells like neutrophils and macrophages. More extended series- and GalNAc-GM1b gangliosides are likely present in the data, however, close isobaric overlap with GD1 species hinders confident identification with MALDI TIMS MS/MS. It is important to note that polysialylated gangliosides are prone to in-source fragmentation. Loss of sialic acid(s) can be observed, resulting in artificially high intensities of monosialylated species and misidentification of in-source fragments as endogenous molecules. While MALDI IMS is a soft ionization technique that can minimize this effect, in-source fragmentation may still be observed. Further optimization of instrument parameters and sample preparation strategies can enhance MALDI IMS of gangliosides.

### Ganglioside Isomers

Monosialylated GM1s can have a single sialic acid on the internal galactose unit (GM1a) or on the terminal galactose unit (GM1b, o-series). Sialic acid diversity is also prevalent, where both NeuAc and NeuGc are common in mammalian non-neuronal cells. Here, MALDI TIMS IMS was used to separate, visualize, and identify GM1 isomers that differ by type and position of the sialic acid. Conformational changes can occur in the gas phase when ions are subjected to prolonged storage and separation times; however, based on previous work by our group, we do not expect conformational changes to play a significant role in GM1 isomer separations, as presented here.^42,49^ All detected GM1 isomers are listed in Tables S8 and S9.

### GM1a and GM1b Isomers

**Figure 4:**
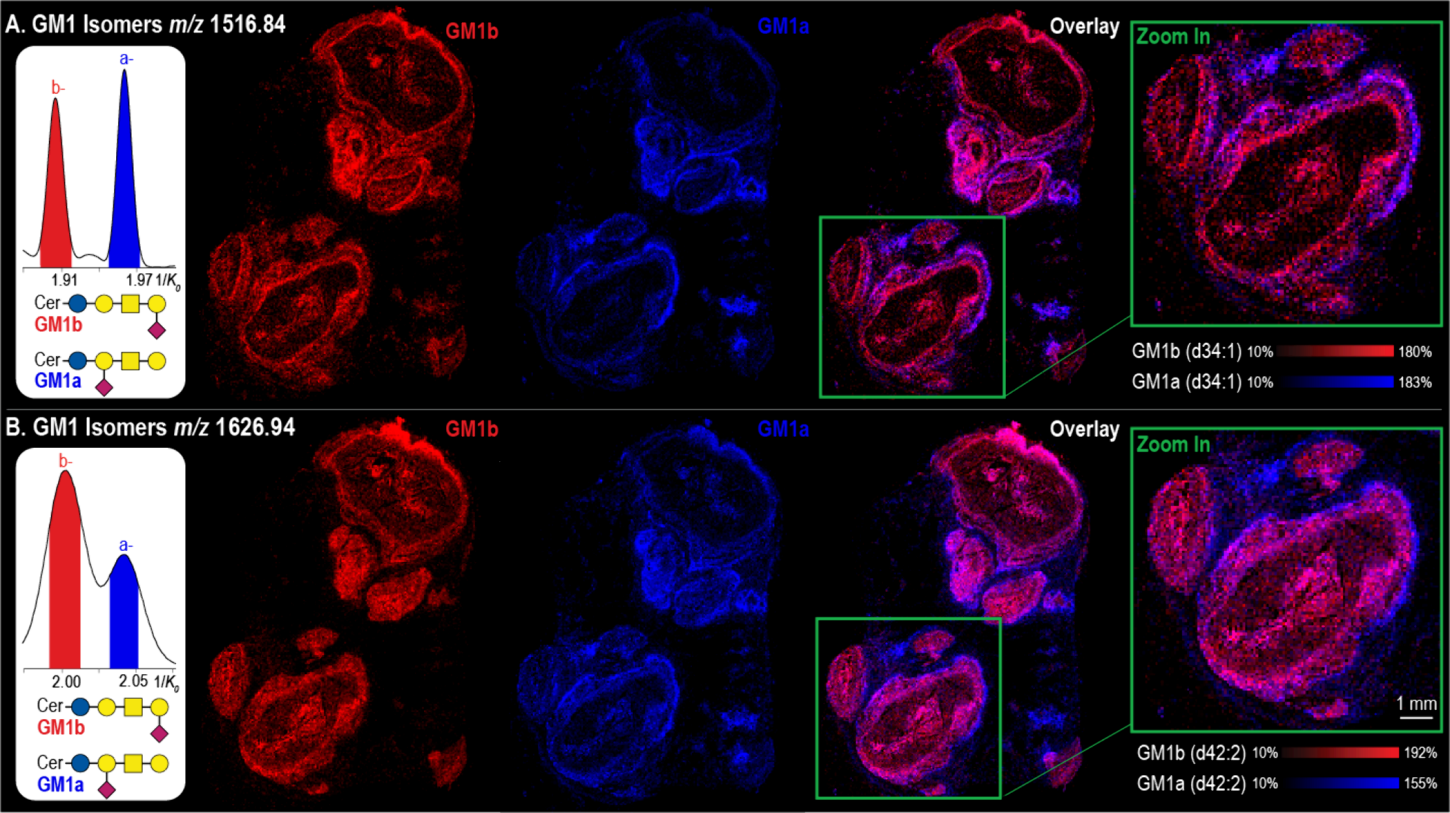
Extracted ion mobilograms of *m*/*z* 1516.84 (A) and *m*/*z* 1626.94 (B) reveal the TIMS separation of GM1b (red) and GM1a (blue) in *S.aureus*-infected mouse kidney section. Ion images of GM1b (red), GM1a (blue), and an overlay of both can be seen for GM1(d34:1) and GM1(d42:2) in (A) and (B), respectively.

All GM1 a- and o-series isomers identified by MALDI TIMS IMS are listed in Table S8. Two examples are highlighted in Figure 4. Here, the extracted ion mobilograms of *m*/*z* 1516.84 (GM1 NeuAc (d34:1)) and *m*/*z* 1626.95 (GM1 NeuAc (d42:2)) revealed two resolved ions (Figure 4A and B). To illustrate the unique spatial distributions of each isomeric species, both the *m*/*z* and 1/*K*_*0*_ information were used to generate the ion images. Each panel highlights the distinct spatial distributions of the lower mobility ion in red, the higher mobility ion in blue, and an overlay of both ion images. In both examples, the more compact structures localized to the internal regions of the abscess, whereas the higher mobility ions localized to the external boundary of the abscess and to regions of immune cell infiltration beyond the defined abscesses. Subsequent on-tissue MALDI TIMS MS/MS collected from a serial tissue section was used to identify the isomers. Example fragmentation data is shown for *m*/*z* 1626.95 (Figure S11). Here, the fragmentation confirmed the ganglioside class (GM1), the type of sialic acid present (NeuAc), the ceramide chain composition (d42:2), and the presence of both GM1b and GM1a isomers (via their preferential/unique fragments). In particular, the fragment ions at *m*/*z* 1217.83 (Cer-Glc-Gal-NeuAC) and *m*/*z* 1173.81 (Cer-Glc-Gal-GalNAc) allowed for the differentiation of a- and o-series isomers, respectively. The extracted ion mobilograms of the fragments were used to link each isomer to its corresponding mobility of the parent ion. It was concluded that the lower mobility ion was GM1b (red), and the higher - GM1a (blue).

### NeuAc-tCer and NeuGc-dCer Ganglioside Isomers

**Figure 5:**
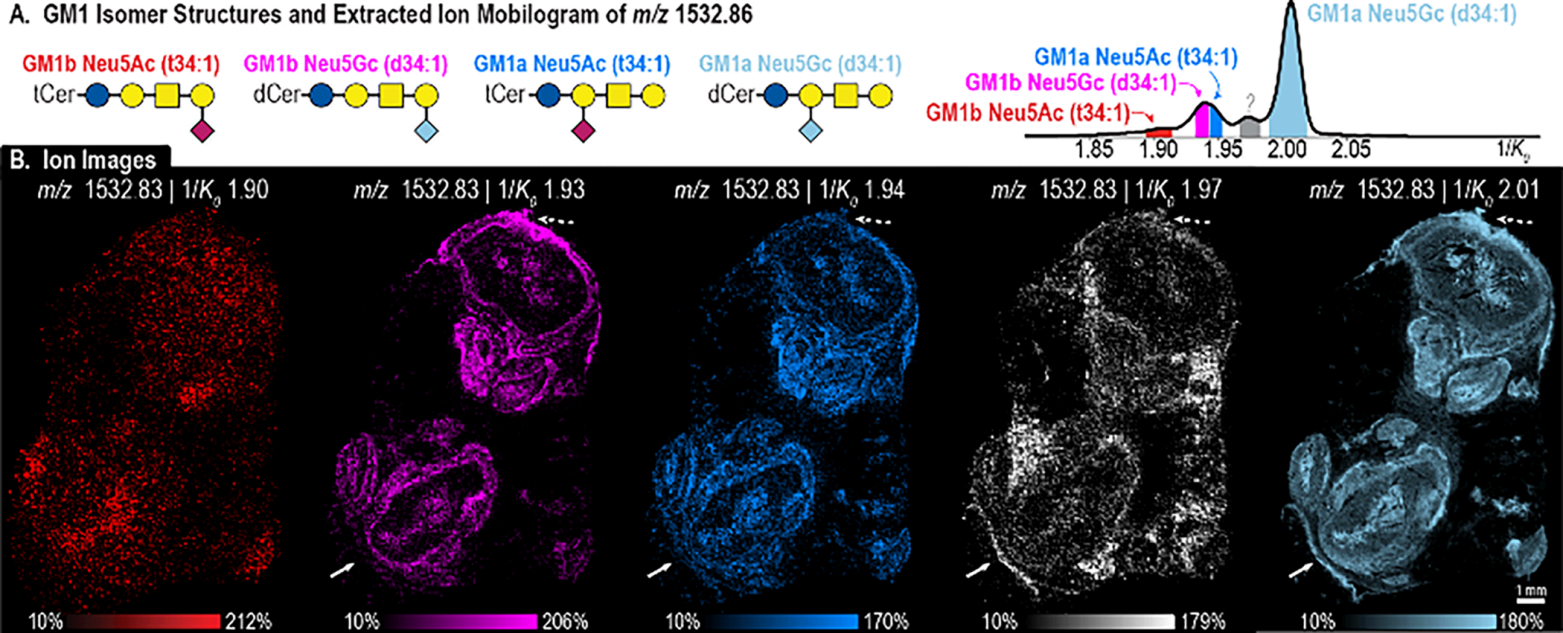
Extracted ion mobilogram of *m*/*z* 1532.83 reveals five partially resolved peaks: 1/*K*_*0*_ 1.90 (red), 1/*K*_*0*_ 1.93 (pink), 1/*K*_*0*_ 1.94 (blue), 1/*K*_*0*_ 1.97 (gray/white), and 1/*K*_*0*_ 2.01 (light blue) (A), where each *m*/*z* + 1/*K*_*0*_ ion distribution can be seen in the ion images (B). Ions were identified with on-tissue MALDI TIMS MS/MS, as seen in Figure 6.

NeuAc-tCer and NeuGc-dCer GM1 isomers arise when a NeuGc-containing GM1 with a dihydroxylated ceramide chain overlaps in mass with a NeuAc sialic acid with a trihydroxylated ceramide chain. Table S9 summarizes the possible NeuGc-dCer and NeuAc-tCer isomers within the data set. Figure 5 highlights GM1 NeuGc (d34:1) and GM1 NeuAc (t34:1), detected at *m*/*z* 1532.83. Additionally, both gangliosides can be GM1a and GM1b, resulting in at least four possible isomers under a single *m*/*z* (Figure S12). The extracted ion mobilogram of *m*/*z* 1532.83 reveals several gas-phase conformers (Figure 5A): 1/*K*_*0*_ 1.90 (red), 1/*K*_*0*_ 1.93 (pink), 1/*K*_*0*_ 1.94 (blue), 1/*K*_*0*_ 1.97 (gray), and 1/*K*_*0*_ 2.01 (light blue). Each of the *m*/*z* + 1/*K*_*0*_ ion distributions can be seen in the ion images in Figure 5B. While all ions localize to the lesions and areas of immune cell infiltration, subtle differences in their spatial distributions can be seen. For example, GM1a NeuGC (d34:1) (green) localizes to the ray-like structure beyond the lower left abscess, denoted with a solid arrow. This isomer is also more intense around the abscess borders and near primary zones of immune cell infiltration. Both GM1b NeuAC (d34:1) (pink) and GM1a NeuGC (d34:1) (light blue) have higher intensities in the outer boundary of the lesion (dotted arrow), where the GM1a NeuAc (t34:1) (blue) is not as prevalent. Finally, the unidentified isomer (white) cannot be seen in the abscesses, but is found throughout the outer cortex of the kidney, possibly indicating areas of immune cell infiltrates, not yet organized into the abscesses.

**Figure 6:**
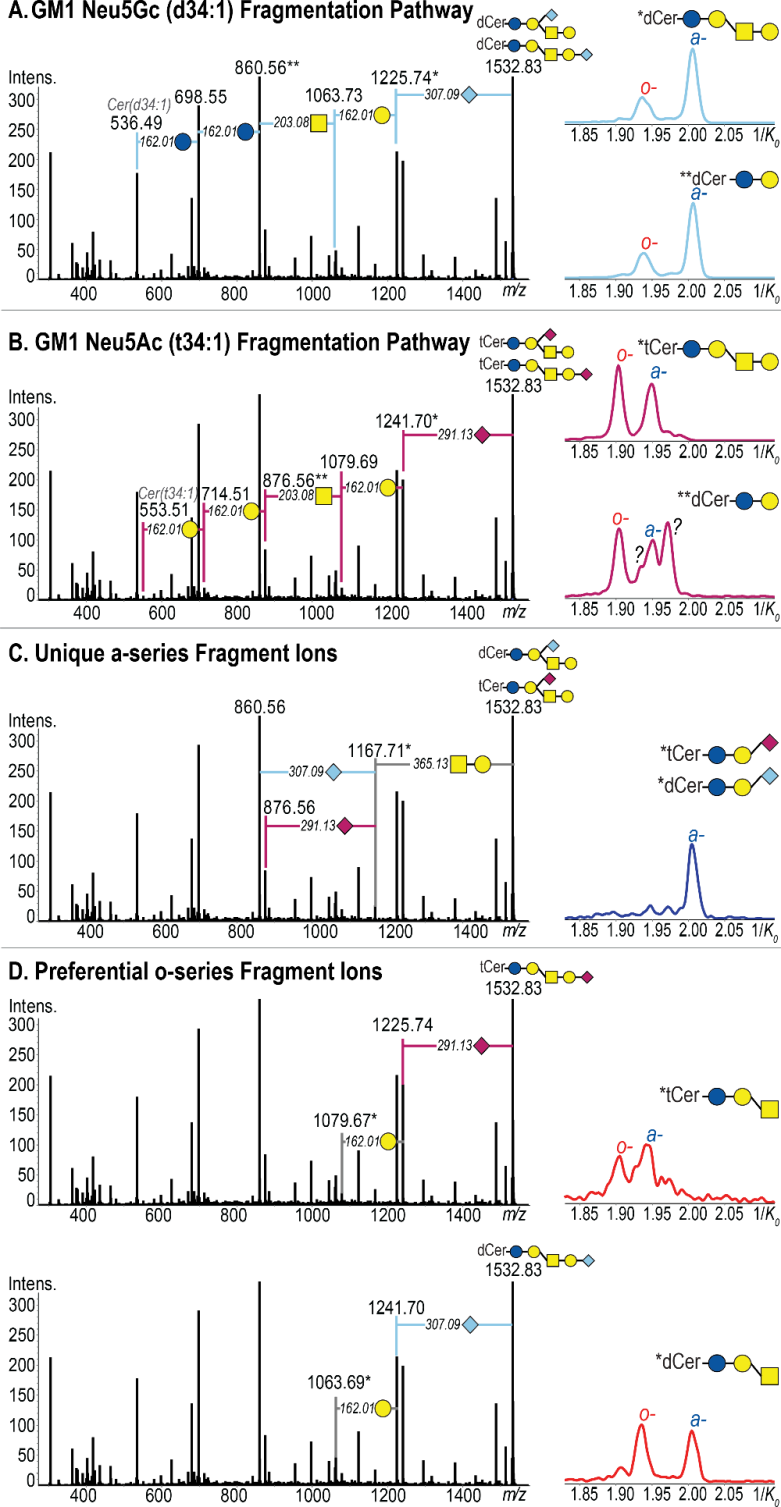
On-tissue MALDI TIMS MS/MS of *m*/*z* 1532.83 reveals the presence of isomeric GM1 Neu5Gc (d34:1) (A) and GM1 Neu5Ac (t34:1) (B) in a 10 DPI *S.aureus*-infected mouse kidney. For both GM1 Neu5Gc (d34:1) and GM1 Neu5Ac (t34:1), GM1a and GM1b isomers were also identified, as evidenced by (C) and (D), respectively.

On-tissue MALDI TIMS MS/MS was performed on a serial tissue section to identify the conformers. The fragmentation spectrum revealed the presence of both GM1 NeuAc (t34:1) and GM1 NeuGc (d34:1), shown in Figure 6A and 6B, respectively. The sialic acid structures were confirmed by the neutral loss of NeuAc (- *m*/*z* 291.13) and NeuGc (- *m*/*z* 307.09). The ceramide compositions Cer(t34:1) and Cer(d34:1) were confirmed by fragments at *m*/*z* 536.49 and *m*/*z* 553.51, respectively (Figure 6A and B). To assign identities of the parent-peak resolved conformers, the extracted ion mobilograms of fragment ions were generated where applicable. The extracted ion mobilograms of *m*/*z* 1225.74 and *m*/*z* 860.56, indicate that GM1 NeuGc (d34:1) is associated with peaks 1/*K*_*0*_ 1.93(1) (pink) and 1/*K*_*0*_ 2.01 (light blue). The presence of two peaks in the extracted fragment ion mobilogram also indicates the presence of both GM1a and GM1b NeuAc (t34:1) isomers. Similarly, the extracted ion mobilograms of *m*/*z* 1241.70 and *m*/*z* 876.56, revealed that the peaks at 1/*K*_*0*_ 1.91 (red) and 1/*K*_*0*_ 1.93(2) (blue) arise from GM1 NeuAc (t34:1) – both GM1a and GM1b isomers. Lastly, the extracted ion mobilogram of *m*/*z* 876.56 revealed two additional isomers, likely arising from ceramide chain diversity, that could not be identified (Figure 6B). To confirm the presence of a- and o-series isomers, unique (or preferential) fragments of each series and their extracted ion mobilograms were studied (Figure 6C and D). The fragment at *m*/*z* 1167.71 is unique for GM1a (both NeuGc (d34:1) and NeuAC (t34:1)). This fragment arises from the loss of Gal-GalNAc from the headgroup, while the sialic acid remains intact on the internal Gal unit.^102^ The extracted ion mobilogram also revealed that NeuAc (t34:1) is the more intense ion. The preferential fragmentation of GM1b gangliosides includes a stepwise loss of a sialic acid, and a Gal unit.^34,102^ For GM1 NeuAc (t34:1) and GM1 NeuGc (d34:1), this results in fragment ions *m*/*z* 1079.67, and *m*/*z* 1063.69, respectively. From the extracted ion mobilogram of each, two peaks can be seen. The lower mobility peaks are attributed to the o-series isomers (GM1b), however, higher-mobility a-series fragmentation was also observed. A more comprehensively annotated fragmentation spectrum is included in the Supporting Information (Figure S13).

Ultimately, we confirmed the presence of four different isomers - NeuAc (t34:1) and NeuGc (d34:1), both a- and o-series of each, and mapped their distinct spatial distributions (Figure 5) within the infection lesion. These findings indicate that the major isomer in the mixture is GM1a NeuAC (t34:1), 1/*K*_*0*_ 2.01; therefore, the molecular image of *m*/*z* 1532.83 would be dominated by this isomer without mobility separation.

## Conclusions

The work described herein investigates ganglioside structural and spatial heterogeneity within a soft tissue *S. aureus* abscess. MALDI TIMS IMS revealed the spatial distributions of gangliosides that varied by class, ceramide chain composition, and sialic acid type and position. While some ions localized to known abscess architecture, others revealed molecular heterogeneity, which could not be discerned by pathohistological assessment. Integrating TIMS allowed for gas-phase separation of GM1b, GM1a, NeuAc-tCer, and NeuGc-dCer isomers within the abscess. This level of structural specificity is essential in elucidating the distinct functions isomeric gangliosides have at the site of infection. For example, previous studies have implicated GM1b isomers and NeuGc-containing gangliosides in host–pathogen interactions; and researchers have postulated that ganglioside patterns of immune cells vary significantly throughout the course of an infection.^55,54,56^ Although significant strides have been made toward understanding the role of gangliosides within infections, much of the knowledge remains descriptive.^18^ Comprehensive analyses, as described herein, are integral to pushing this research beyond characterization. Gaining fundamental insight into the metabolism of gangliosides, and glycosphingolipids as a whole,^18^ is a promising avenue of research for potential treatment and/or prevention of infectious diseases.

## Supporting Information Available

Ganglioside synthesis pathway; average mass spectrum of control and infected mouse kidney section; ion mobility heat map of 10 DPI mouse kidney average mass spectrum; table summarizing matrix deposition parameters; table summarizing characteristic fragment ions used for ganglioside identification; tables and figures of gangliosides identified in a 10 DPI mouse kidney section, including GM3, GM2, GM1, GalNAc- and extended series GM1b, and GD1; on-tissue fragmentation of GM2 and GalNAc-GM1b; tables listing ganglioside isomers identified in 10 DPI mouse kidney, including GM1a and GM1b, and NeuAc-tCer and NeuGc-dCer; on-tissue fragmentation of a- and o-series ganglioside isomers (*m*/*z* 1626.95); molecular structures of GM1 isomers detected at *m*/*z* 1532.83; detailed on-tissue fragmentation of *m*/*z* 1532.83 (PDF)
