# Supplementary material for: MALDI TIMS IMS Reveals Ganglioside Molecular Diversity within Murine S. aureus Kidney Tissue Abscesses
Source: J Am Soc Mass Spectrom. 2024 Jul 25;35(8):1692–701. doi: 10.1021/jasms.4c00089 (PMC11311236; doi:10.1021/jasms.4c00089)
Supplement: Supplementary file 1 — js4c00089_si_001.pdf [file js4c00089_si_001.pdf]

MALDI TIMS IMS Reveals Ganglioside Molecular Diversity within Murine *S. aureus* Kidney Tissue Abscesses.

Katerina V. Djambazova[1,2], Katherine N. Gibson-Corley[3], Jeffrey A. Freiberg[4,5], Richard M. Caprioli[2,3,6-9], Eric P. Skaar[3,4,10], and Jeffrey M. Spraggins[1-3,6,9]*.

[1]Department of Cell and Developmental Biology, Vanderbilt University, Nashville, TN 37232, USA
[2]Mass Spectrometry Research Center, Vanderbilt University, Nashville, TN 37232, USA
[3]Department of Pathology, Microbiology, and Immunology, Vanderbilt University Medical Center, Nashville, TN 37232, USA
[4]Vanderbilt Institute for Infection, Immunology and Inflammation, Vanderbilt University Medical Center, Nashville, TN 37232, USA
[5]Division of Infectious Diseases, Department of Medicine, Vanderbilt University Medical Center, Nashville, TN 37232, USA
[6]Department of Biochemistry, Vanderbilt University, Nashville, TN 37232, USA
[7]Department of Pharmacology, Vanderbilt University, Nashville, TN 37232, USA
[8]Department of Medicine, Vanderbilt University, Nashville, TN 37232, USA
[9]Department of Chemistry, Vanderbilt University, Nashville, TN 37232, USA
[10]Vanderbilt Institute for Chemical Biology, Vanderbilt University, Nashville, TN 37232, USA

CORRESPONDING AUTHOR: Jeffrey M. Spraggins: jeff.spraggins@vanderbilt.edu

---

SUPPORTING INFORMATION

Table of Contents

Figure S1	Ganglioside Synthesis Pathway….…………...…………………………………..…………….….....	Page S-2
Figure S2	Average Mass Spectra of Control and 10 DPI Mouse Kidney Sections………………….…………...	Page S-3
Figure S3	Ion Mobility Heat Map of 10 DPI Mouse Kidney Section……..……………….…………………..…	Page S-3
Table S1	MALDI IMS Matrix Deposition Parameters…………………………………………….…..……...	Page S-3
Table S2	Characteristic Fragment Ion for on-tissue Ganglioside identification..……………………...…..……..	Page S-4
Table S3	GM3 Gangliosides Identified in a 10 DPI Mouse Kidney Section……..……………………...…..…	Page S-4
Figure S4	Example GM3 ion images in control and 10DPI Mouse Kidney Section…….………….………..…	Page S-5
Table S4	GM2 Gangliosides Identified in a 10 DPI Mouse Kidney Section ……………………………..……...	Page S-5
Figure S5	Example GM2 ion images in control and 10DPI Mouse Kidney Section…….………….……......…	Page S-6
Figure S6	On-tissue Fragmentation of GM2 NeuGC (d34:1)……………………………………...…..…….	Page S-6
Table S5	GM1 Gangliosides Identified in a 10 DPI Mouse Kidney Section ……………………………..……...	Page S-7
Figure S7	Example GM1 ion images in control and 10DPI Mouse Kidney Section…….……………..……...	Page S-8
Table S6	GD1 Gangliosides Identified in a 10 DPI Mouse Kidney Section ……………………………..……...	Page S-8
Figure S8	Example GD1 ion images in control and 10DPI Mouse Kidney Section…….…………….…..………	Page S-9
Table S7	GalNAc-GM1b and Extended Series GM1b Identified in a 10 DPI Mouse Kidney Section.. …...…….	Page S-10
Figure S9	Example GalNAc-GM1b and extended series GM1b ion images in control and 10DPI Mouse Kidney Section…….…………...…………...…………...…………...…………...…………...…………………	Page S-11
Figure S10	On-tissue Fragmentation of GalNAc-GM1b (d42:1) ………………………………………….….……..	Page S-12
Table S8	GM1a and GM1b Isomers Identified in a 10 DPI Mouse Kidney Section……………………….….…...	Page S-12

Table S9	NeuAc-tCer and NeuGc-dCer Isomers Identified in a 10 DPI Mouse Kidney Section ..…...……..…...	Page S-12
Figure S11	On-tissue Fragmentation of GM1a and GM1b Isomers detected at *m/z* 1626.95……………..….…….	Page S-13
Figure S12	Molecular Structures of GM1 Isomers Detected at *m/z* 1532.83………………………………..….…...	Page S-14
Figure S13	Detailed On-tissue Fragmentation of *m/z* 1532.83………...……………….……………...…..…...	Page S-15

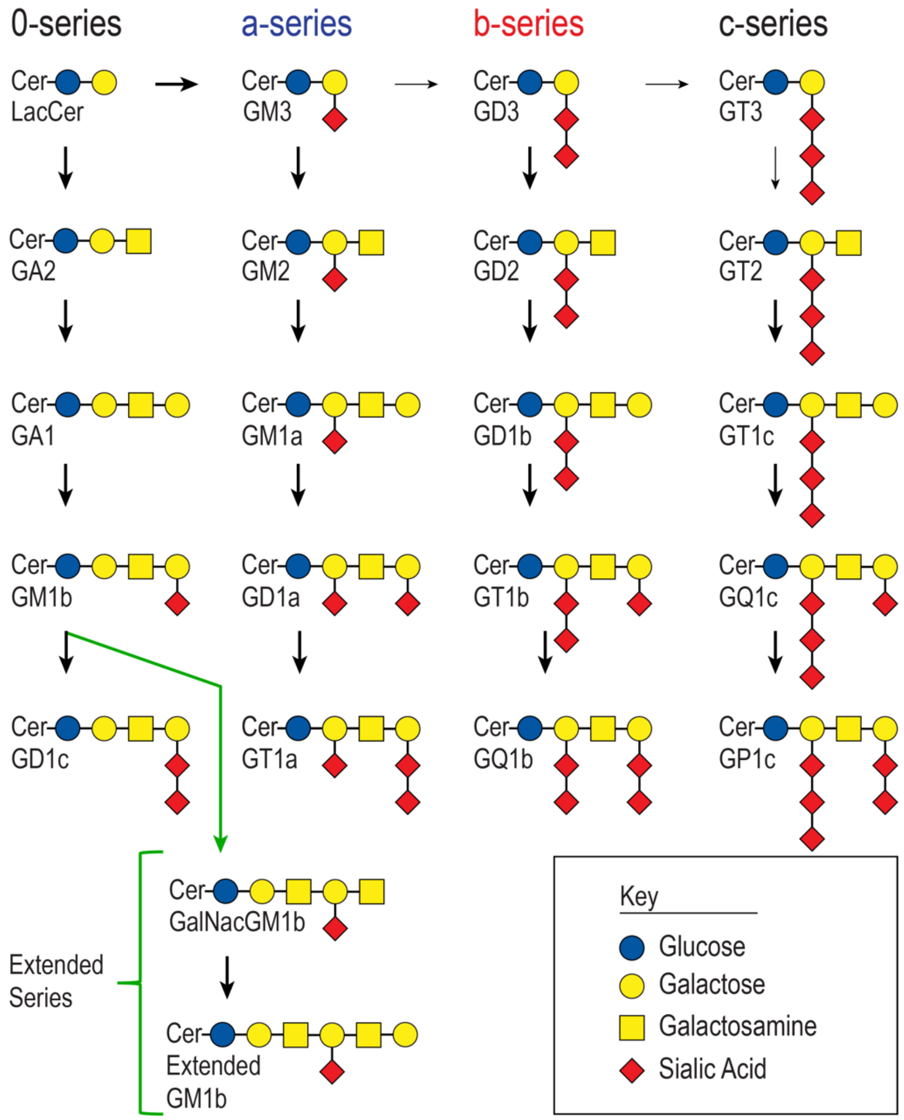

Figure S1. Ganglioside synthesis pathway highlighting major gangliosides found in mammalian cells, including extended series gangliosides, known to be involved in the immune system.

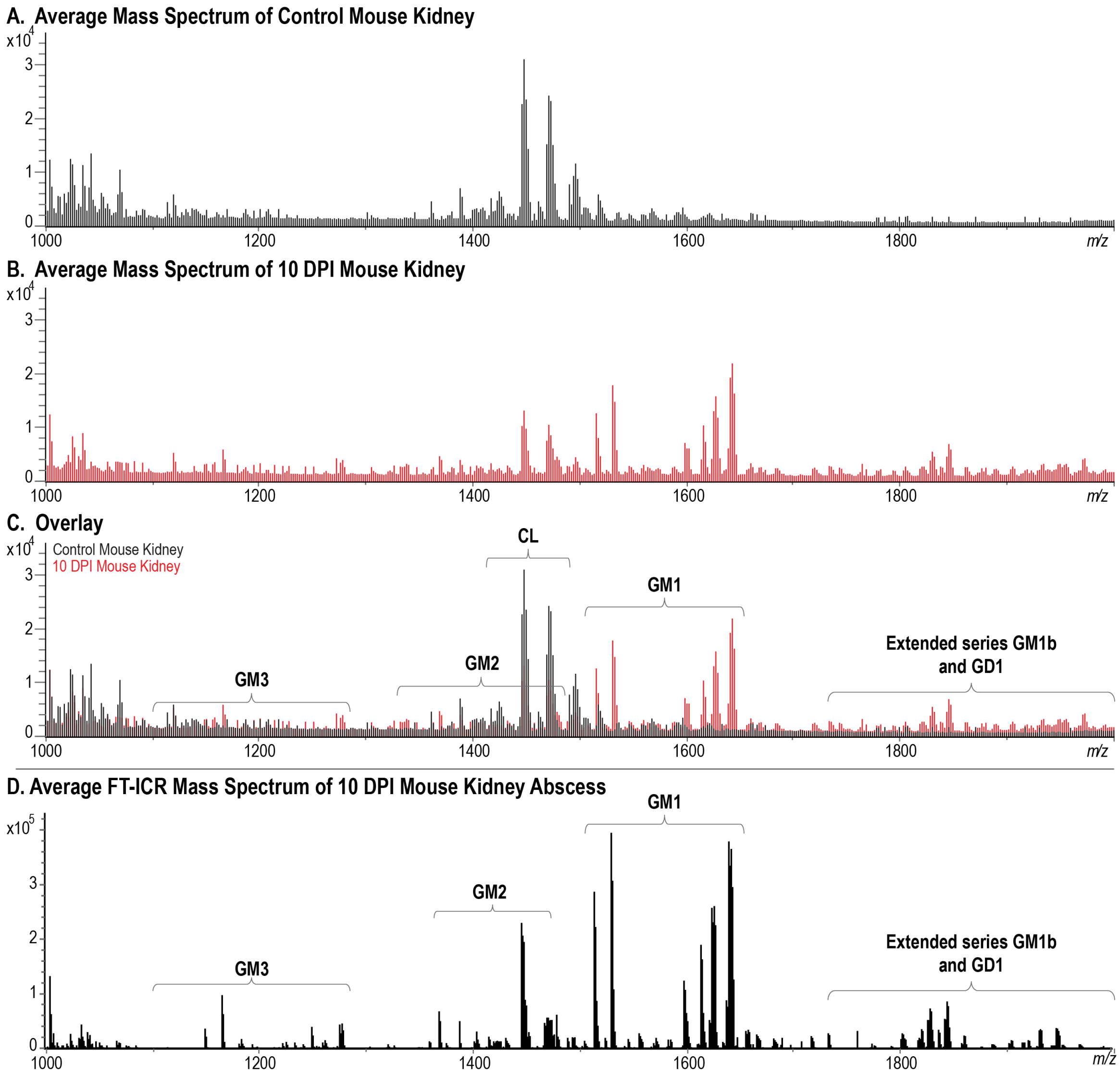

Figure S2. Average MALDI TIMS mass spectrum of control **(A)** and 10 DPI *S. aureus*-infected **(B)** mouse kidney tissue section and an overlay of both spectra **(C)**. Average FT-ICR mass spectrum of the abscessed region, collected from a serial section for high mass accuracy ganglioside identification **(D)**. Major ganglioside classes detected in the infected kidney are identified in the overlay.

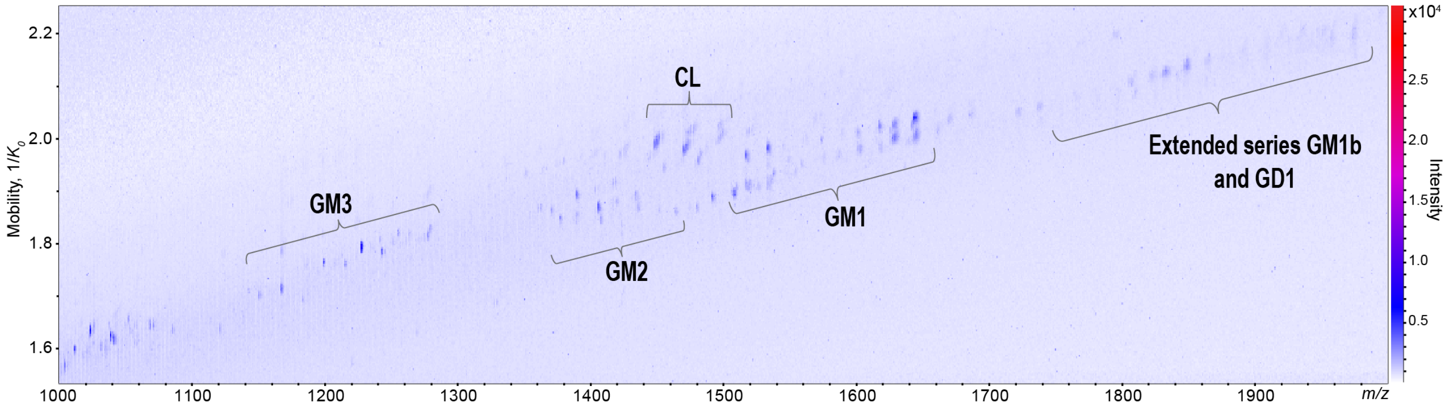

Figure S3. Ion mobility heat map of average mass spectrum of 10 DPI *S. aureus*-infected mouse kidney tissue section.

Table S1. Matrix Deposition Parameters for Ganglioside Analysis within Murine Kidney Infection Model

	Concentration (mg/mL), Solvent	Nozzle/Stage temperature (°C)	Velocity (m/min)	Passes (#)	Spray Speed (mL/min)	Matrix density (µg/mm^2)
DHA-AmS	5.0, 62.5 mM AmS, 60% Ethanol	60/50	1350	5	0.05, 10 psi	1.48

Nozzle height (40 mm), spacing (1.5 mm), gas flow rate (2 L/min), and drying time (2 sec).

Table S2. Characteristic Fragment Ion and Neutral loss used for ganglioside species Identification

m/z		Fragment Ion	Fragment type	Elemental composition
162.01	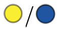	Gal or Glc	Neutral Loss	$C_6H_{10}O_5$
203.06	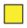	GalNAc	Neutral Loss	$C_8H_{13}NO_5$
365.13	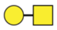	Gal-GalNAc	Neutral Loss	$C_{14}H_{23}NO_{10}$
291.13	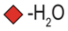	Neu5Ac – H_2O	Neutral Loss	$C_{11}H_{17}NO_8$
307.09	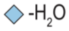	Neu5Gc – H_2O	Neutral Loss	$C_{11}H_{17}NO_9$
567.20	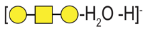	[GalNAc-Gal-GalNAc $-3H_2O-H]^-$	Fragment ion	$C_{22}H_{35}N_2O_{15}$

This is not an exhaustive list of diagnostic fragments used in ganglioside identification; it only reflects fragment ions and neutral losses observed and used in this study.

Table S3. GM3 Gangliosides Identified in a 10 DPI Mouse Kidney Section; identified.

Ganglioside	Full Name	*Theoretical m/z*	*Experimental m/z*	*ppm Error*	*FT experimental m/z*	*ppm Error*
GM3 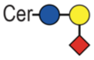	GM3 NeuAc (d34:1)*	1151.70586	1151.7106	-4.12	1151.70716	-1.13
	GM3 NeuGc (d34:1)	1167.70078	1167.7060	-4.47	1167.70225	-1.26
	GM3 NeuGc (t34:1)	1183.69569	1183.6992	-2.97	1183.69665	-0.81
	GM3 NeuGc (d38:1)*	1223.76334	1223.7659	-2.09	1223.76501	-1.36
	GM3 NeuAc (d40:1)*	1235.79976	1235.8038	-3.27	1235.80113	-1.11
	GM3 NeuGc (d40:1)*	1251.79468	1251.7984	-2.97	1251.79591	-0.98
	GM3 NeuAc (d42:2)*	1261.81541	1261.8191	-2.92	1261.81740	-1.58
	GM3 NeuAc (d42:1)*	1263.83106	1263.8333	-1.77	1263.83296	-1.50
	GM3 NeuGc (d42:3)	1275.79468	1275.7962	-1.19	1275.79572	-0.82
	GM3 NeuGc (d42:2)*	1277.81033	1277.8135	-2.48	1277.81138	-0.82
	GM3 NeuGc (d42:1)*	1279.82598	1279.8279	-1.50	1279.82834	-1.84

*Denotes the ganglioside species that were also detected in the control mouse kidney. Ion images of these species can be seen in Figure S4.

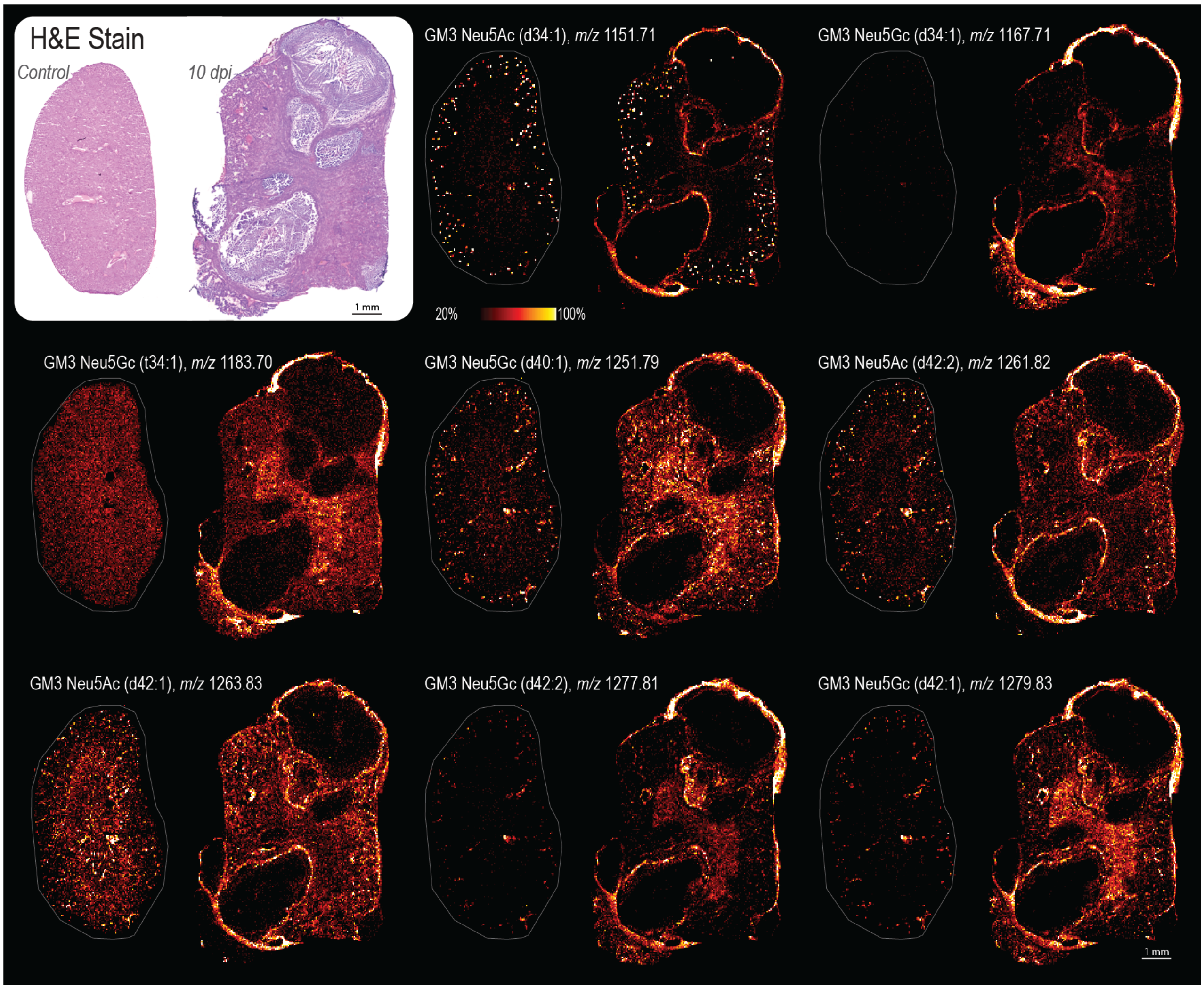

Figure S4. Example ion images of eight GM3 gangliosides in control and 10DPI *S. aureus*-infected mouse kidney tissue sections. GM3s were the only class of gangliosides detected above the limit of detection in the control samples.

Table S4. GM2 Gangliosides Identified in a 10 DPI Mouse Kidney Section

Ganglioside	Full Name	*Theoretical m/z*	*timsTOF Experimental m/z*	*ppm Error*	*FT Experimental m/z*	*ppm Error*
GM2 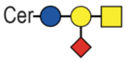	GM2 NeuGc (d34:1)	1370.78016	1370.7830	-2.07	1370.78097	-0.59
	GM2 NeuGc (d40:1)	1454.87406	1454.8757	-1.13	1454.87345	0.42
	GM2 NeuGc (d42:2)	1480.88971	1480.8912	-1.01	1480.89141	-1.15
	GM2 NeuGc (d42:1)	1482.90536	1482.9033	1.39	1482.90613	-0.52

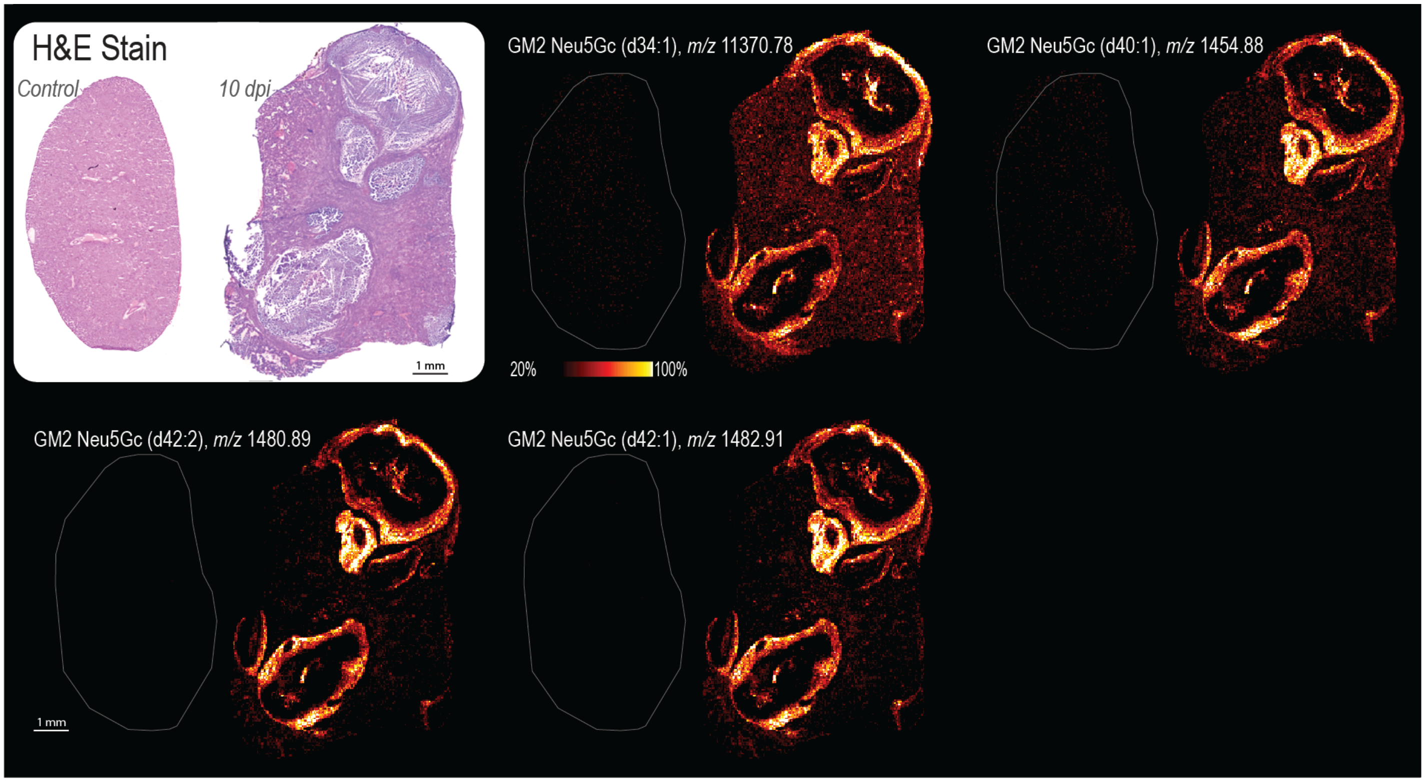

Figure S5. Example ion images of seven GM2 gangliosides in control and 10DPI *S. aureus*-infected mouse kidney tissue sections.

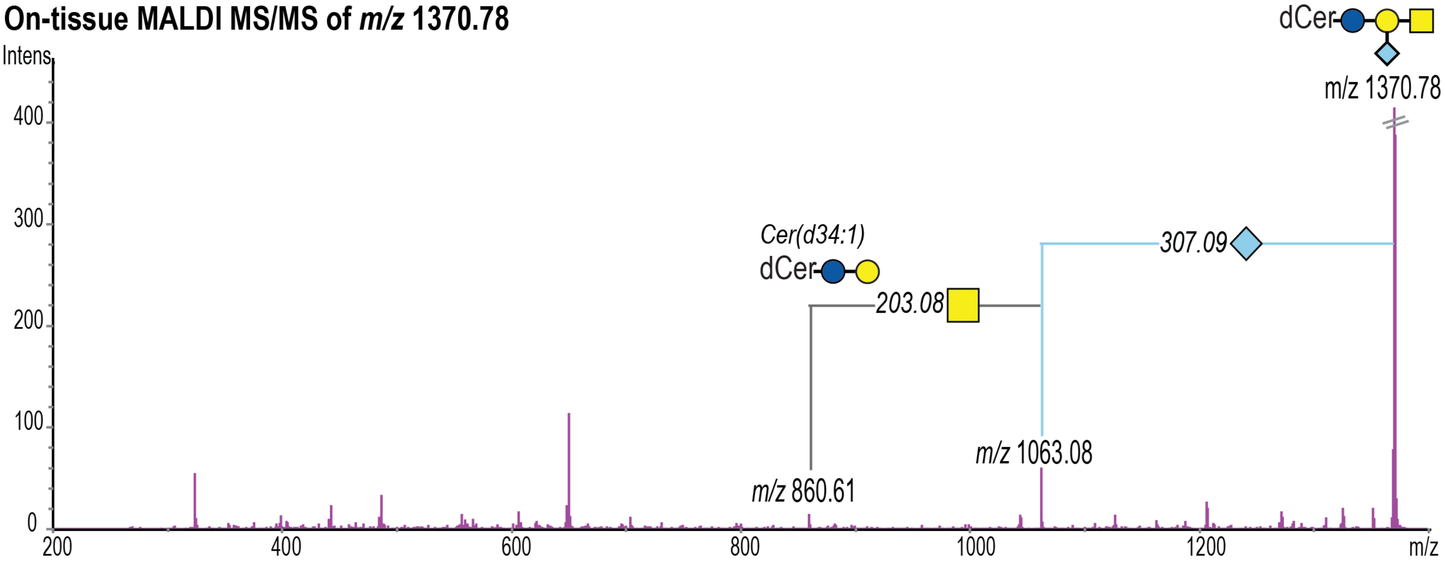

Figure S6. On-tissue MALDI MS/MS of *m/z* 1370.78 reveals the structure of GM2 NeuGC (d34:1). The peak at *m/z* 1063.08 indicates the neutral loss of sialic acid (NeuGC – H_2O), *m/z* 860.61 confirms the ceramide composition as (d34:1). No neutral loss of NeuAC sialic acid (-291.09) was observed in the fragmentation spectrum, indicating Neu5GC-containing GM2 was the most likely the only isomer with *m/z* 1370.78. The absolute intensity of *m/z* 1370.78 is 1964. MALDI TIMS MS/MS IMS data were collected in negative ionization mode with the following parameters – isolation mass: *m/z* 1370.78 ± 1.5, CID: 55.0 eV, *m/z* range: *m/z* 200 – 3000, area of data collection: 285 pixels.

Table S5. GM1 Gangliosides Identified in a 10 DPI Mouse Kidney Section

Ganglioside	Full Name	*Theoretical m/z*	*timsTOF Experimental m/z*	*ppm Error*	*FT Experimental m/z*	*ppm Error*
GM1 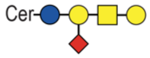	GM1 NeuAc (d34:1)	1516.83806	1516.8400	-1.28	1516.83899	-0.61
	GM1 NeuAc (d34:0)	1518.85371	1518.8493	2.90	1518.84707	4.37
	GM1 NeuGc (d34:1)	1532.83298	1532.8345	-0.99	1532.83507	-1.36
	GM1 NeuAc (t34:1)	1532.83298	1532.8345	-0.99	1532.83507	-1.36
	GM1 NeuAc (d36:1)	1544.86936	1544.8696	-0.16	1544.87102	-1.07
	GM1 NeuGc (d36:1)	1560.86428	1560.8626	1.08	1560.86712	-1.82
	GM1 NeuAc (t36:1)	1560.86426	1560.8626	1.06	1560.86712	-1.83
	GM1 NeuAc (t38:1)	1588.89556	1588.8970	-0.91	1588.89689	-0.84
	GM1 NeuGc (d38:1)	1588.89556	1588.8970	-0.91	1588.89689	-0.84
	GM1 NeuAc (t38:0)	1590.91120	1590.9018	5.91	1590.90587	3.35
	GM1 NeuAc (d40:1)	1600.93196	1600.9335	-0.96	1600.93251	-0.34
	GM1 NeuAc (d40:0)	1602.94760	1602.9445	1.93	1602.94740	0.12
	GM1 NeuGc (d40:1)	1616.92686	1616.9281	-0.77	1616.92929	-1.50
	GM1 NeuAc (t40:1)	1616.92686	1616.9281	-0.77	1616.92929	-1.50
	GM1 NeuAc (t40:0)	1618.9425	1618.9373	3.21	1618.93641	3.76
	GM1 NeuAc (d42:3)	1624.93196	1624.9324	-0.27	1624.93204	-0.05
	GM1 NeuAc (d42:2)	1626.94761	1626.9479	-0.18	1626.94969	-1.28
	GM1 NeuAc (d42:1)	1628.96326	1628.9614	1.14	1628.96575	-1.53
	GM1 NeuGc (t42:3)	1640.92688	1640.9273	-0.26	1640.92899	-1.29
	GM1 NeuGc (d42:2)	1642.94251	1642.9427	-0.12	1642.94554	-1.84
	GM1 NeuAc (t42:2)	1642.94251	1642.9427	-0.12	1642.94554	-1.84
	GM1 NeuGc (d42:1)	1644.95818	1644.9561	1.26	1644.95929	-0.67
	GM1 NeuAc (t42:1)	1644.95818	1644.9561	1.26	1644.95929	-0.67

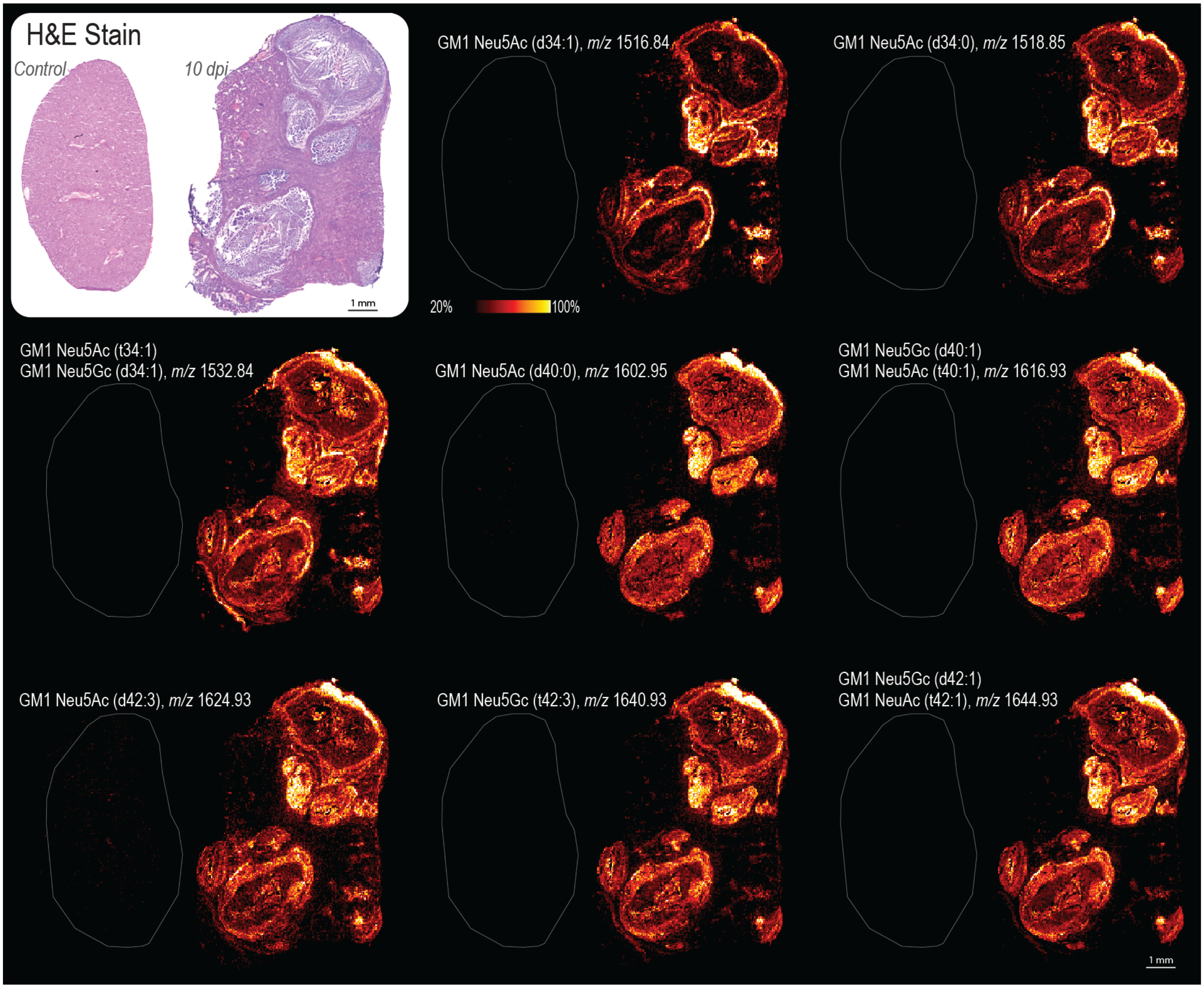

Figure S7. Example ion images of seven GM1 gangliosides in control and 10 DPI *S. aureus*-infected mouse kidney tissue sections.

Table S6. GD1 Gangliosides Identified in a 10 DPI Mouse Kidney Section; *Denotes potential isomers that were not identified.

Ganglioside	Full Name	*Theoretical m/z*	*timsTOF Experimental m/z*	*ppm Error*	*FT Experimental m/z*	*ppm Error*
GD1 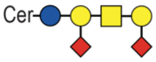	GD1 (NeuAc - NeuAc) (d34:1)	1807.93347	1807.9345	-0.57	1807.93426	-0.44
	GD1 (34:1)*	1823.92839	1823.9290	-0.33	1823.92925	-0.47
	GD1 (34:1)*	1839.92330	1839.9238	-0.27	1839.92736	-2.21
	GD1 (40:1)*	1908.02229	1908.0433	-11.01	1908.02983	-3.95
	GD1 (NeuGc - NeuGc) (d40:1)	1924.01720	1924.0190	-0.94	1924.01425	1.53
	GD1 (42:3)*	1932.02229	1932.0175	2.48	1932.02550	-1.66
	GD1 (42:2)*	1934.03794	1934.0366	0.69	1934.04354	-2.90
	GD1 (42:1)*	1936.05359	1936.0494	2.16	1936.05812	-2.34
	GD1 (42:2)*	1950.03285	1950.0321	0.38	1950.04003	-3.68
	GD1 (42:1)*	1952.04850	1952.0445	2.05	1952.05189	-1.74

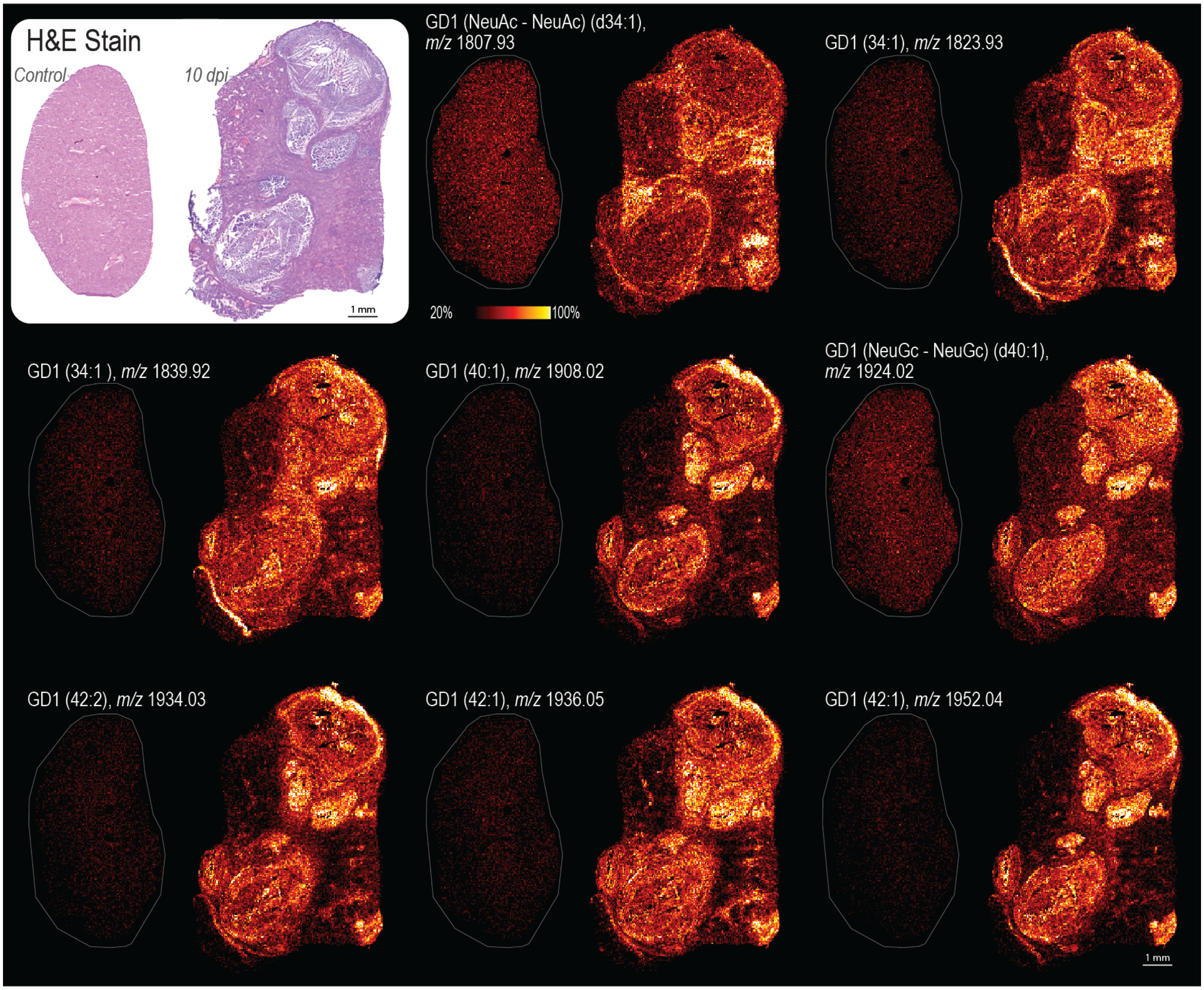

Figure S8. Example ion images of seven GD1 gangliosides in control and 10 DPI *S. aureus*-infected mouse kidney tissue sections.

Table S7. GalNAc-GM1b and extended series GM1b Gangliosides Identified in a 10 DPI Mouse Kidney Section; *Denotes potential isomers that were not identified.

Ganglioside	Full Name	*Theoretical m/z*	*timsTOF Experimental m/z*	*ppm Error*	*FT Experimental m/z*	*ppm Error*
GalNAc-GM1b	GalNAc-GM1b NeuAc (d34:1)	1719.91744	1719.9175	-0.06	1719.92142	-2.31
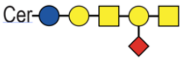	GalNAc-GM1b NeuAc (d34:0)	1721.93309	1721.9288	2.49	1721.93550	-1.40
	GalNAc-GM1b (34:1) *	1735.91236	1735.9124	-0.06	1735.91537	-1.73
	GalNAc-GM1b (34:0)*	1737.92801	1737.9215	3.75	1737.92900	-0.57
	GalNAc-GM1b (36:1)*	1763.94366	1763.9411	1.47	1763.94670	-1.72
	GalNAc-GM1b NeuAc (d40:1)	1804.01134	1804.0111	0.11	1804.01543	-2.27
	GalNAc-GM1b NeuAc (d40:0)	1806.02700	1806.0239	1.72	1806.03040	-1.88
	GalNAc-GM1b (40:1)*	1820.00626	1820.0065	-0.11	1820.00544	0.45
	GalNAc-GM1b (40:0)*	1822.02191	1822.0183	1.98	1822.01876	1.73
	GalNAc-GM1b NeuAc (d42:2)	1830.02699	1830.0259	0.60	1830.03073	-2.04
	GalNAc-GM1b NeuAc (d42:1)	1832.04264	1832.0397	1.58	1832.04627	-1.98
	GalNAc-GM1b (42:3)*	1844.00626	1844.0055	0.43	1844.00737	-0.60
	GalNAc-GM1b (42:2)*	1846.02191	1846.0211	0.43	1846.02110	0.44
	GalNAc-GM1b (42:1)*	1848.03754	1848.0350	1.41	1848.03454	1.62
	GalNAc-GM1b NeuGc (t42:2)	1862.01681	1862.0167	0.05	1862.02485	-4.32
	GalNAc-GM1b NeuGc (t42:1)	1864.03246	1864.0304	1.13	1864.03733	-2.61
Extended GM1b	Ext. GM1b NeuAc (d42:2)	1992.07981	1992.0669	6.48	1992.08698	-3.60
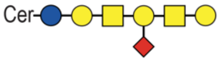	Ext. GM1b NeuAc (d42:1)	1994.09546	1994.0860	4.76	1994.10045	-2.50
	Ext. GM1b NeuGc (42:2)*	2010.09038	2010.0859	2.24	2010.09706	-3.32

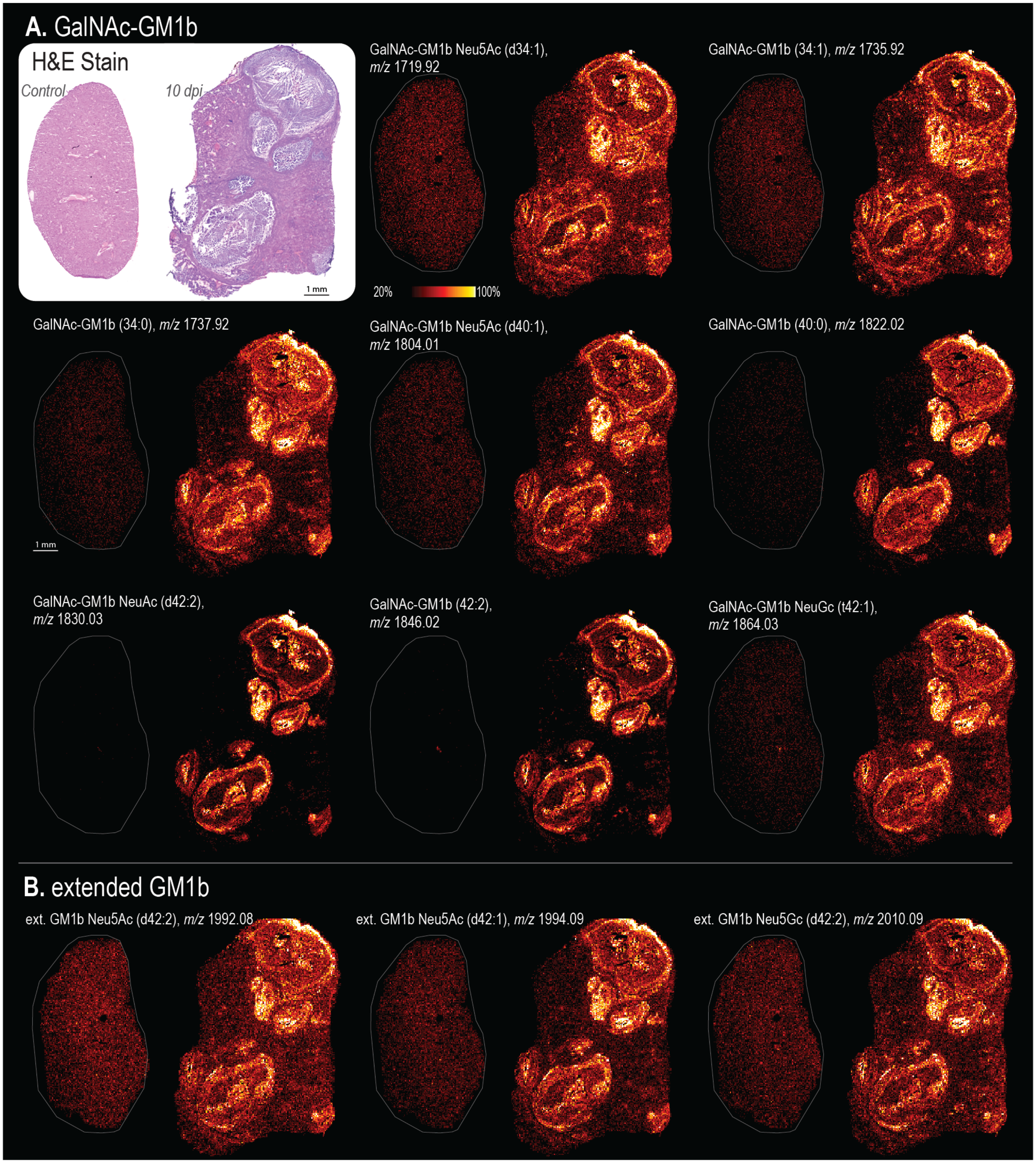

Figure S9. Example ion images of seven GalNAc-GM1b gangliosides **(A)** and three extended series GM1b gangliosides **(B)** in control and 10 DPI *S. aureus*-infected mouse kidney tissue sections.

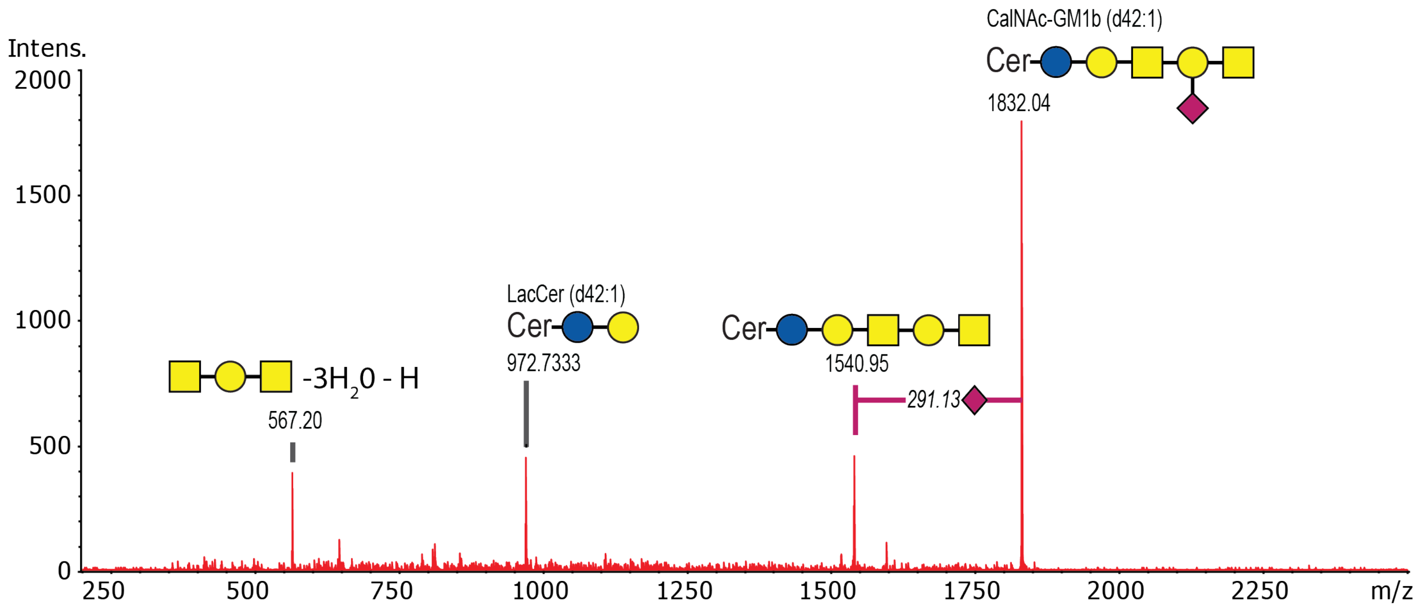

Figure S10. On-tissue MALDI MS/MS of *m/z* 1832.04 reveals the structure of GalNAc-GM1b (d42:1). Data were collected in negative ionization mode with the following parameters: isolation mass: *m/z* 1832.04 ± 1.5, CID: 90.0 eV, *m/z* range: *m/z* 200 – 3000; sum of 10 MS/MS spectra. The peak at *m/z* 1540.95 indicates the neutral loss of sialic acid (NeuAC – H_2O), *m/z* 972.73 confirms the ceramide composition as (d42:1), and *m/z* 567.20 reveals the diagnostic fragment ion of (GalNAc-Gal-GalNAc)-3H_2O-H. These fragmentation ions are consistent with literature (Li et al. J. Chromatogr. A., 2022; 1676(463196)).

Table S8. GM1a and GM1b Ganglioside Isomers Identified in a 10 DPI Mouse Kidney Section

Ganglioside	Full Name	*Theoretical m/z*	*timsTOF Experimental m/z*	*ppm Error*	*FT Experimental m/z*	*ppm Error*
**GM1a and GM1b**	GM1 NeuAc GM1 (d34:1)	1516.83806	1516.8400	-1.28	1516.83899	-0.61
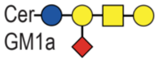	GM1 NeuAc GM1 (d36:1)	1544.86936	1544.8696	-0.16	1544.87102	-1.07
	GM1 NeuAc GM1 (d40:1)	1560.86428	1560.8626	1.08	1560.86712	-1.82
	GM1 NeuAc GM1 (d40:0)	1602.94760	1602.9445	1.93	1602.94740	0.12
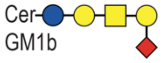	GM1 NeuAc GM1 (d42:3)	1624.93196	1624.9324	-0.27	1624.93204	-0.05
	GM1 NeuAc GM1 (d42:2)	1626.94761	1626.9479	-0.18	1626.94969	-1.28
	GM1 NeuAc GM1 (d42:1)	1628.96326	1628.9614	1.14	1628.96575	-1.53

Table S9. GM1 NeuAc-tCer and NeuGc-dCer Ganglioside Isomers Identified in a 10 DPI Mouse Kidney Section

Ganglioside	Full Name	*Theoretical m/z*	*timsTOF Experimental m/z*	*ppm Error*	*FT Experimental m/z*	*ppm Error*
**GM1**	GM1 NeuGc (d34:1) | GM1 NeuAc (t34:1)	1532.83298	1532.8345	-0.99	1532.83507	-1.36
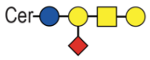	GM1 NeuGc (d36:1) | GM1 NeuAc (t36:1)	1560.86428	1560.8626	1.08	1560.86712	-1.82
	GM1 NeuAc (t38:1) | GM1 NeuGc (d38:1)	1588.89556	1588.8970	-0.91	1588.89689	-0.84
	GM1 NeuGc (d40:1) | GM1 NeuAc (t40:1)	1616.92686	1616.9281	-0.77	1616.92929	-1.50
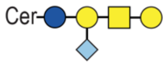	GM1 NeuGc (d40:0) | GM1 NeuAc (t40:0)	1618.94250	1618.9373	3.21	1618.93641	3.76
	GM1 NeuGc (d42:3) | GM1 NeuAc (t42:3)	1640.92688	1640.9273	-0.26	1640.92899	-1.29
a- & o-	GM1 NeuGc (d42:2) | GM1 NeuAc (t42:2)	1642.94251	1642.9427	-0.12	1642.94554	-1.84
isomers possible	GM1 NeuGc (d42:1) | GM1 NeuAc (t42:1)	1644.95818	1644.9561	1.26	1644.95929	-0.67

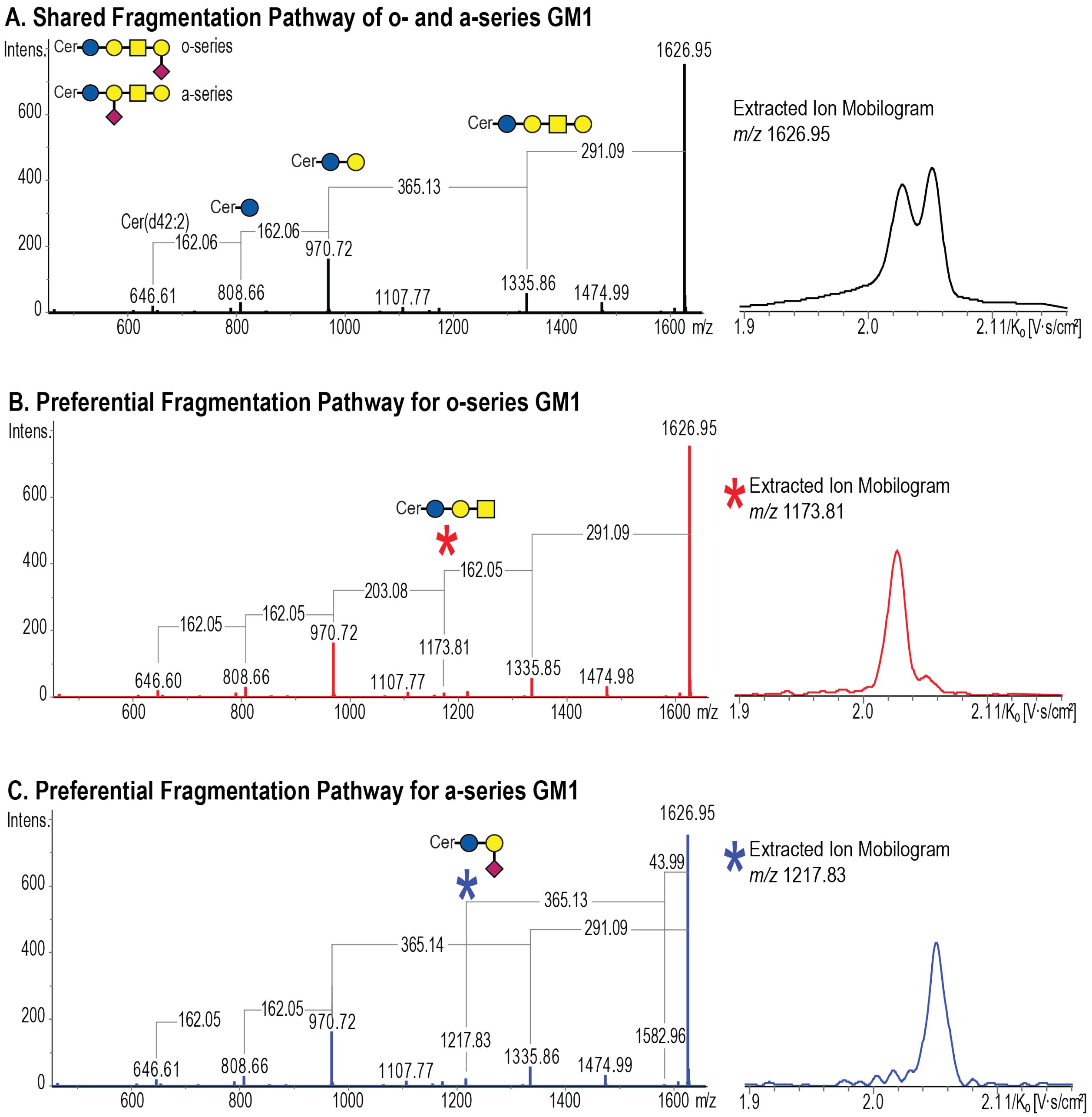

Figure S11. On-tissue MALDI TIMS MS/MS of *m/z* 1626.95 **(A)** reveals the presence of both GM1a **(B)** and GM1b **(C)** isomers within a 10 DPI *S.aureus*-infected mouse kidney. The Extracted ion mobilograms of unique/preferential fragments confirm that GM1b (red) is more compact than GM1a (blue).

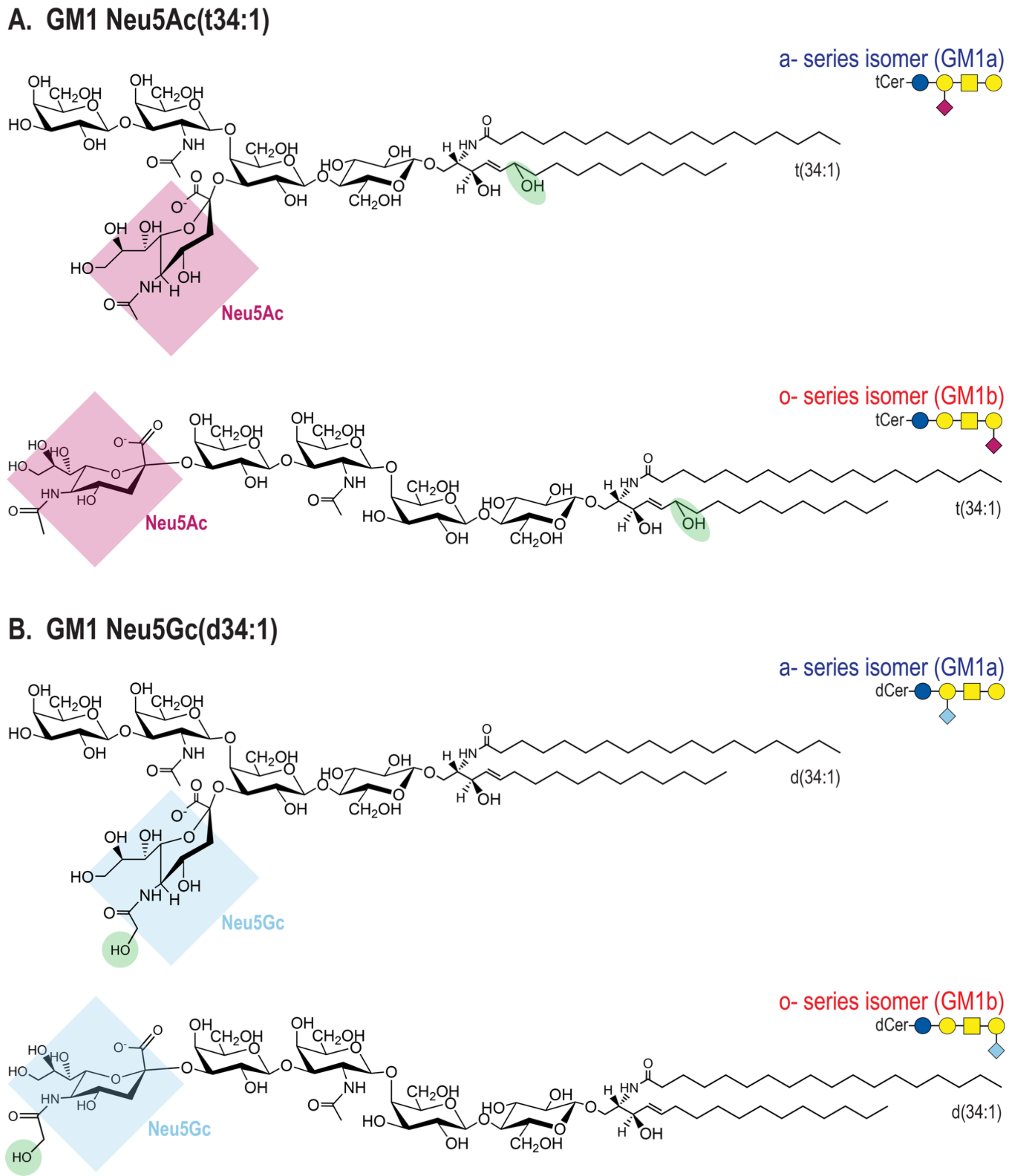

Figure S12. Molecular structures of GM1 isomers, detected at *m/z* 1532.83 include GM1a NeuAc(t34:1) and GM1b NeuAc(t34:1) **(A)**; and GM1a NeuGc(d34:1) and GM1b NeuGc(d34:1) **(B)**.

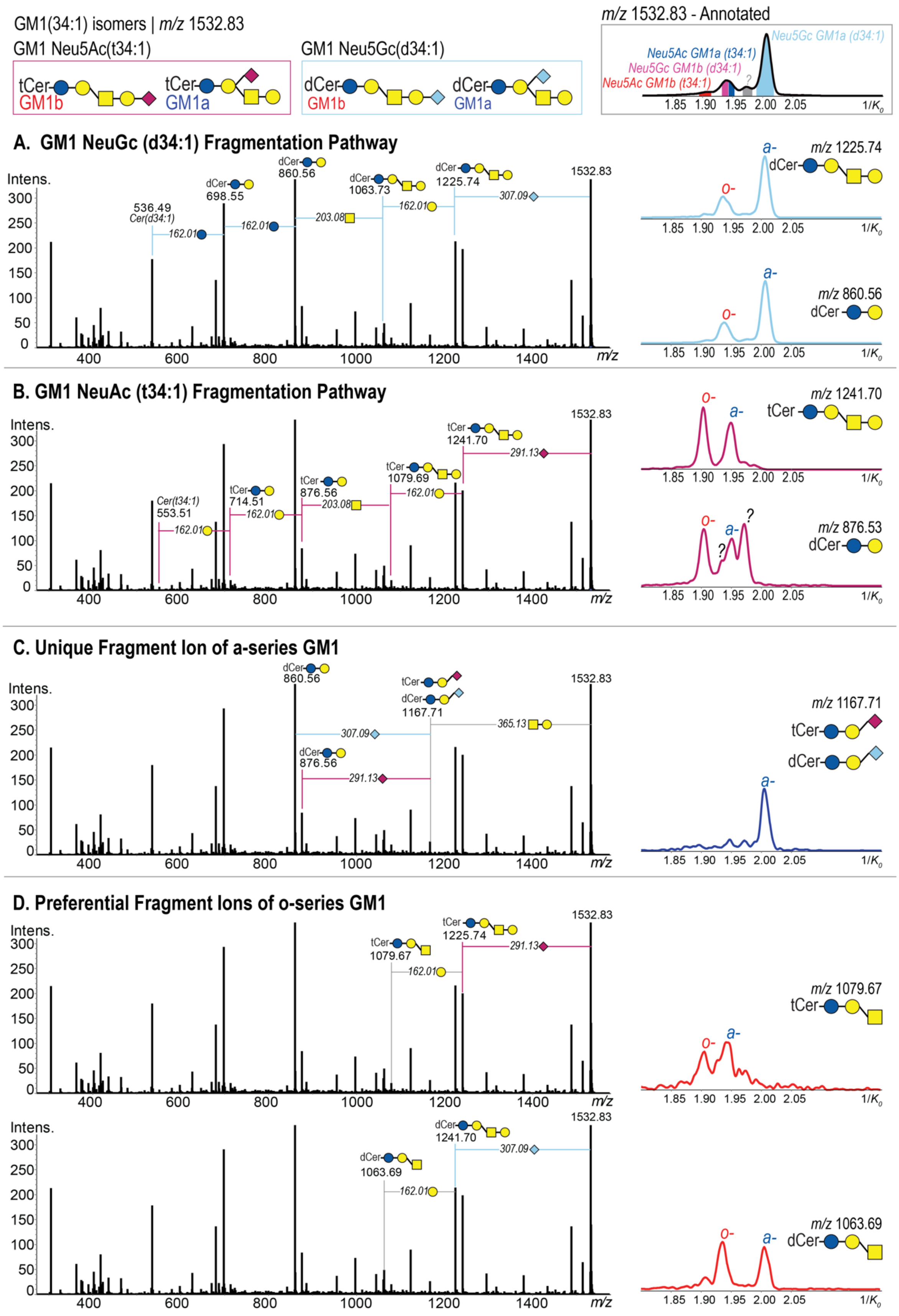

Figure S13. MALDI TIMS on-tissue fragmentation of *m/z* 1532.83 reveals isomeric GM1 NeuGc(d34:1) **(A)** and GM1 NeuAc(t34:1) **(B)**. a- **(C)** and o-series **(D)** isomers of both GM1 NeuGc(d34:1) and GM1 NeuAc(t34:1) were also detected. MALDI TIMS MS/MS IMS data were collected in negative ionization mode, with the following parameters – isolation mass: *m/z* 1532.83 ± 1.5, CID: 85.0 eV, m/z range: *m/z* 100 – 3000, $1/K_0$ range: 0.50 – 2.40 Vs/cm^2, TIMS ramp time: 500 ms, pixels: 5537.
